# Supplementary material for: Glycine Substitution at Helix-to-Coil Transitions Facilitates the Structural Determination of a Stabilized Subtype C HIV Envelope Glycoprotein
Source: Immunity. 2017 May 16;46(5):792–803.e3. doi: 10.1016/j.immuni.2017.04.014 (PMC5439057; doi:10.1016/j.immuni.2017.04.014)
Supplement: Document S1. Figures S1–S6 and Table S1 [file mmc1.pdf]

Immunity, Volume *46*

Supplemental Information

Glycine Substitution at Helix-to-Coil Transitions Facilitates the Structural Determination of a Stabilized Subtype C HIV Envelope Glycoprotein

Javier Guenaga, Fernando Garces, Natalia de Val, Robyn L. Stanfield, Viktoriya Dubrovskaya, Brett Higgins, Barbara Carrette, Andrew B. Ward, Ian A. Wilson, and Richard T. Wyatt

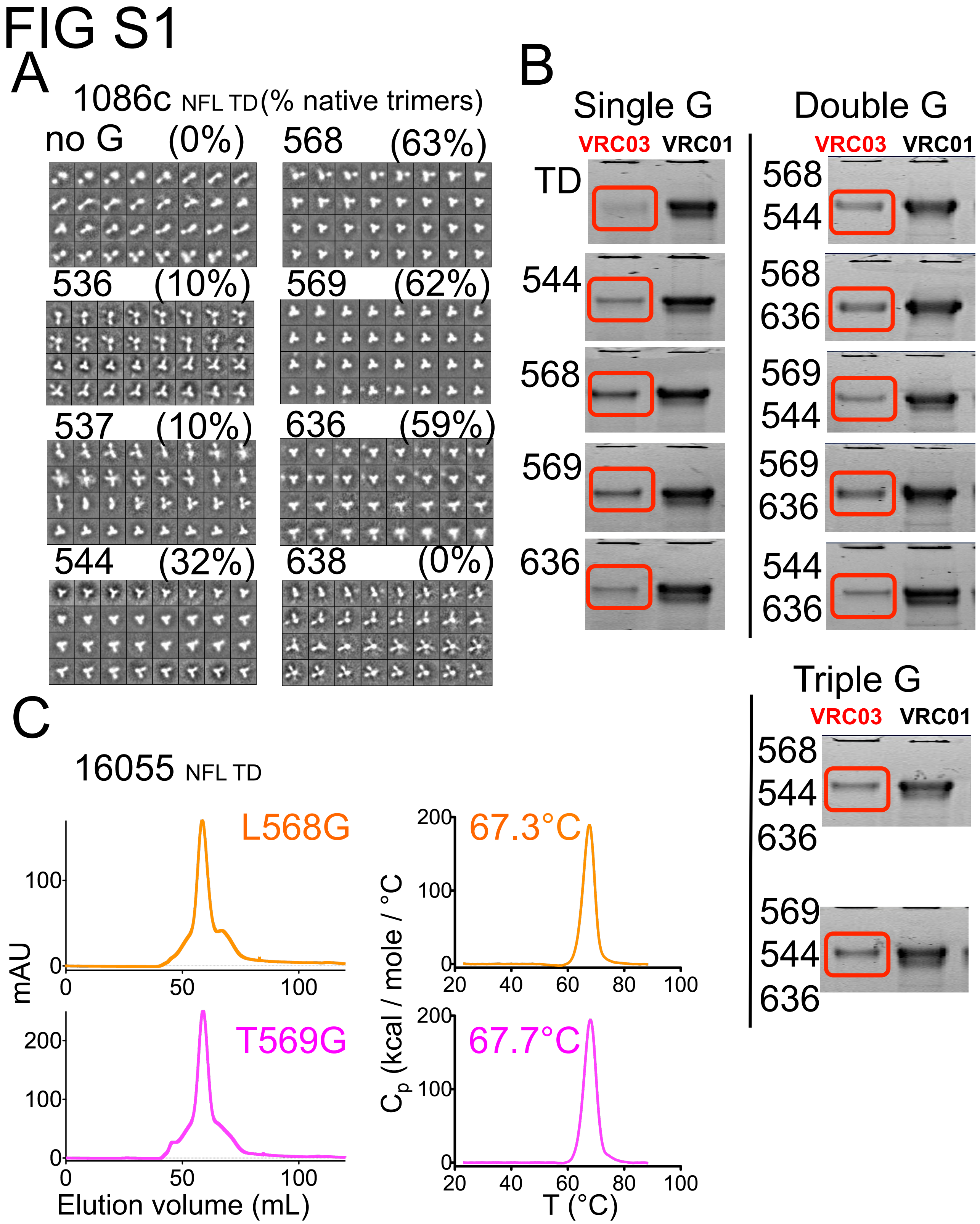

Figure S1. Effect of glycine substitutions on NFL TD proteins. Related to Figure 1. (**A**) EM negative-stain 2D class averages of 1086c NFL TD trimer variants carrying selected gp41 glycine substitutions. The EM-derived frequencies of native-like forms of the trimer variants are shown as percentages of the total purified protein. (**B**) SDS-gel of 1086c NFL TD proteins immunoprecipitated with the trimer-independent CD4bs bNAb VRC01 to control for expression and with the trimer-preferring bNAb VRC03 to indicate the presence of native-like, well-ordered trimers from cell supernatants containing the transiently expressed trimer variants possessing the indicated glycine substitutions.(**C**) SEC and DSC profiles corresponding to the 16055 NFL TD proteins with glycine substitutions at positions 568 and 569.

FIG S2

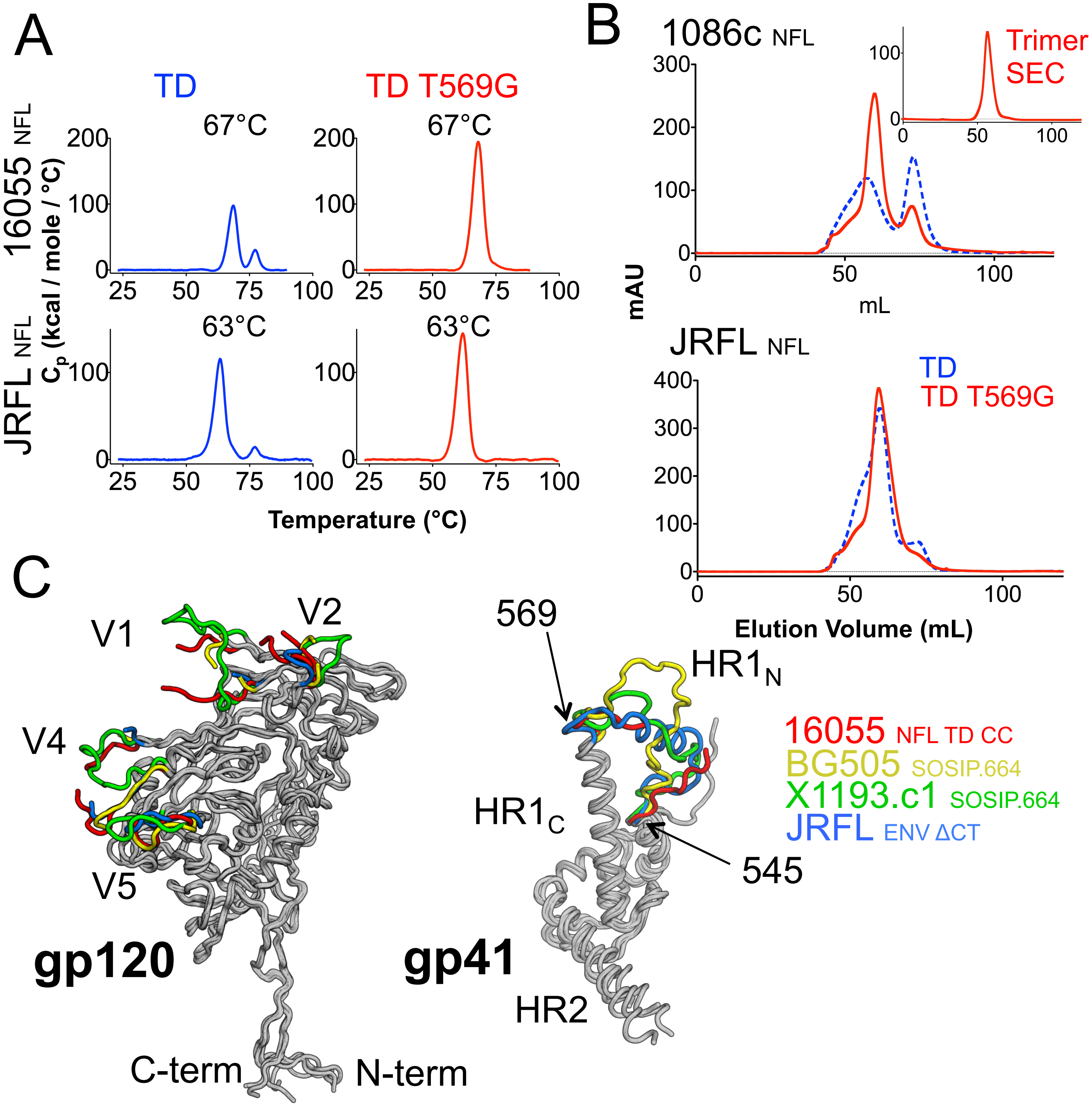

Figure S2. Stability and homogeneity of NFL TD proteins with the T569G mutation and Env regions with structural variation. Related to Figures 1 and 2. (**A**) DSC thermal transition curves of the 16055 and JRFL NFL TD trimers with and without the T569G mutation. (**B**) SEC profiles of HIV-1 subtype C- and B-derived 1086c NFL TD and JRFL NFL TD trimers with and without the T569G mutation. Shown in the upper right is the SEC profile corresponding to a re-run of the trimer fractions. (**C**) Superimposition of structures of Env subunits corresponding to four different HIV strains with differences highlighted in color [(16055, red), (BG505, yellow PDB 5CEZ),(X1193.c1, green PDB 5FYJ), and (JRFL, blue PDB 5FUU)].

FIG S3

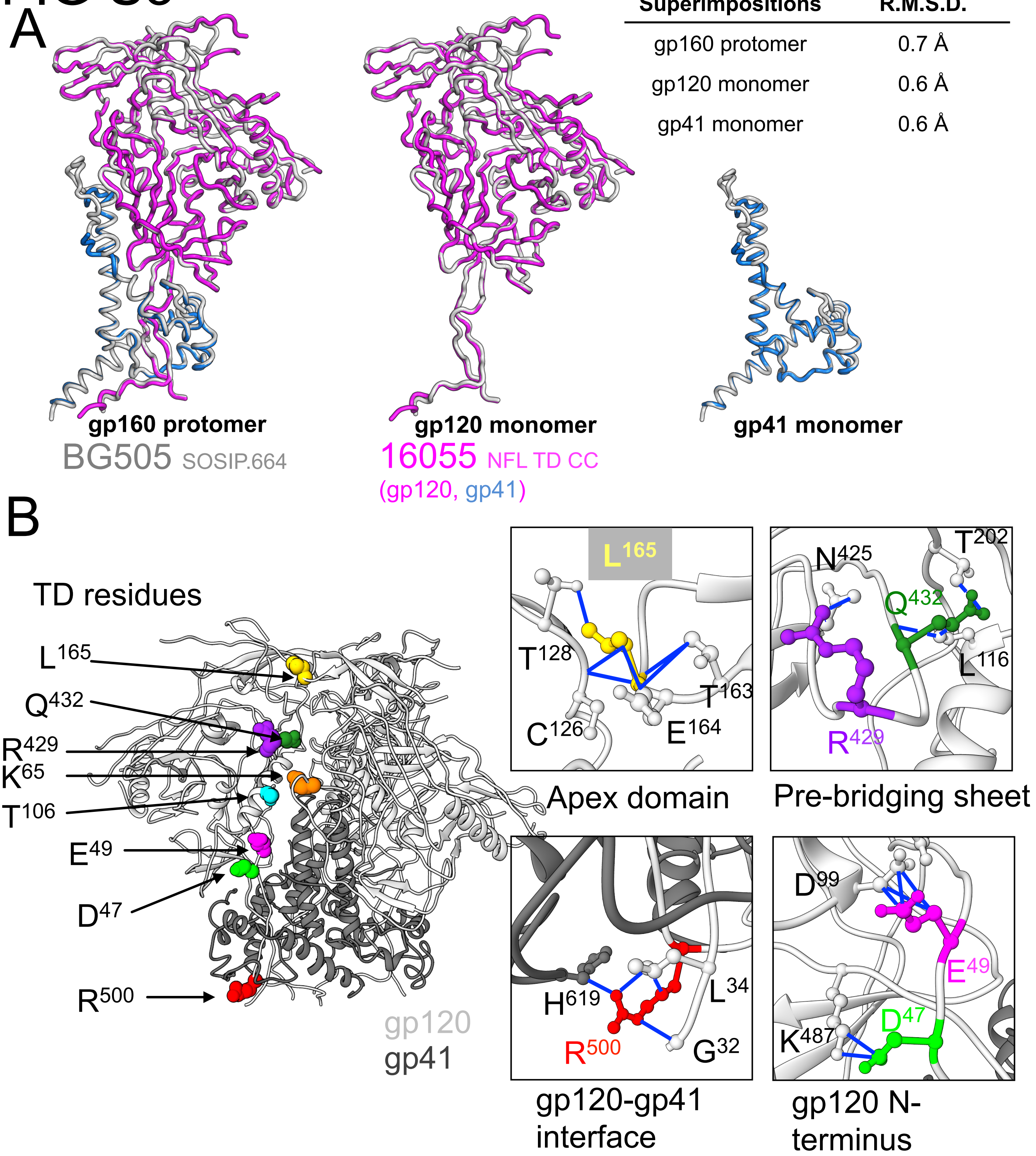

Figure S3. Structural analysis of 16055 NFL TD CC (T569G). Related to Figure 2. (**A**) Superimposition of the gp160 protomers (left), gp120 subunits (middle), and gp41 subunits (right) from the PGT124/35O22-bound 16055 and the PGT121/35O22-bound BG505 crystal structures with Cα RMSD measurements. (**B**) Cartoon representation of the 16055 NFL TD CC trimer with TD residues represented as colored spheres (left). Close-up view of specific TD residues and their contacts (right).

FIG S4

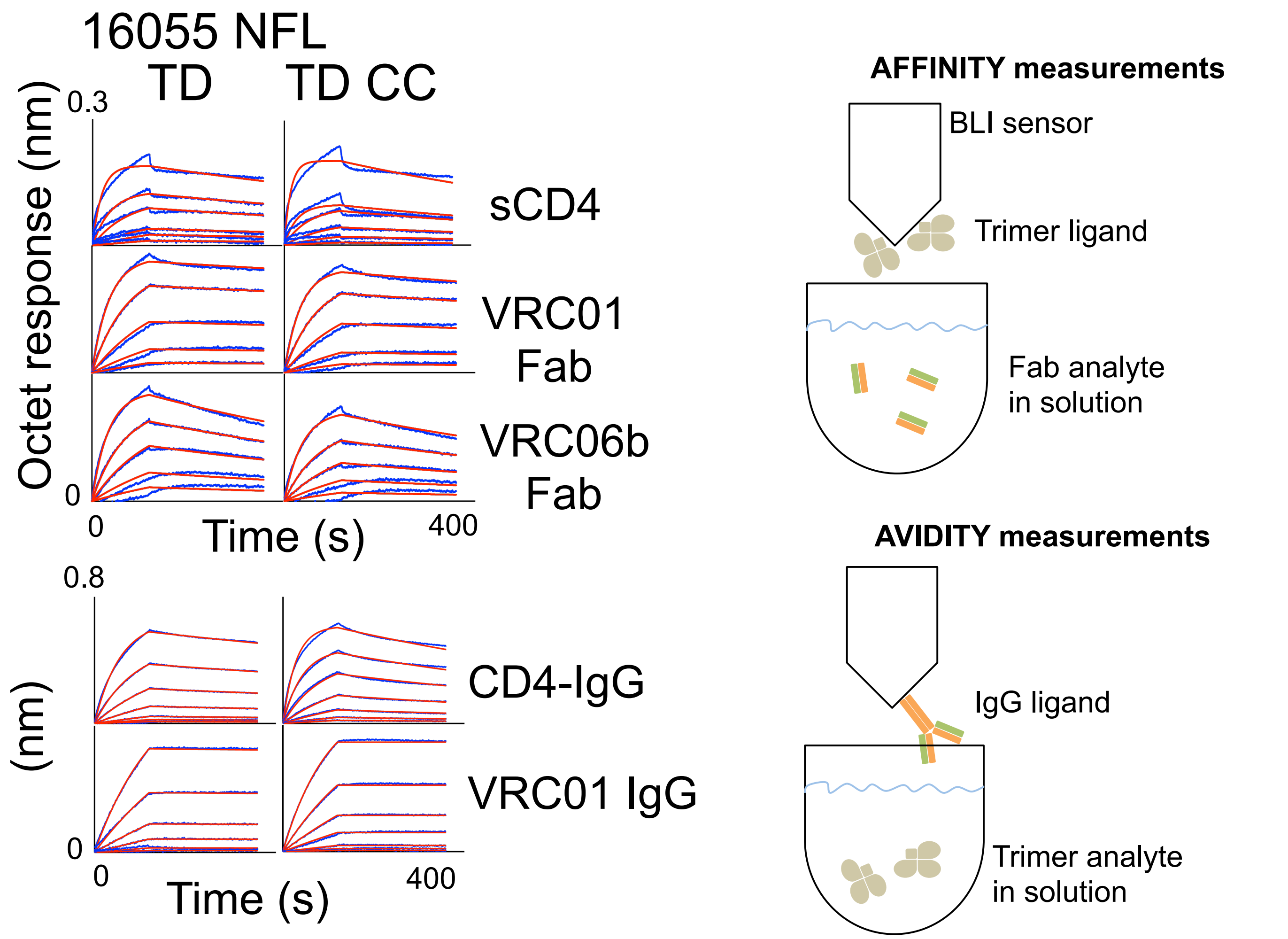

Analyte	Ligand	K_D (nM)	k_{on} (1/Ms)	k_{off} (1/s)	
sCD4	TD	54	1.6×10^4	8.7×10^{-4}	AFFINITY
	TD CC	54	2.2×10^4	1.2×10^{-3}	
VRC01 Fab	TD	30	8.5×10^3	2.5×10^{-4}	
	TD CC	44	9.0×10^3	4.0×10^{-4}	
VRC06b Fab	TD	270	4.4×10^3	1.2×10^{-3}	
	TD CC	282	4.0×10^3	1.1×10^{-3}	
Trimer TD	CD4-IgG	25	2.2×10^4	5.5×10^{-4}	AVIDITY
Trimer TD CC		25	4.3×10^4	1.1×10^{-3}	
Trimer TD	VRC01 IgG	<1	8.9×10^3	$<1 \times 10^{-5}$	
Trimer TD CC		<1	1.1×10^4	$<1 \times 10^{-5}$	

Figure S4. BLI measurements of selected CD4bs ligands and NFL trimers. Related to Figure 5. BLI binding curves (blue) and Langmuir (1:1) model fits (red) for the interaction of monomeric analytes (four-domain-sCD4, VRC01 and VRC03 Fabs) with trimeric ligands, 16055 NFL TD (T569G) with and without 201-433CC. Avidity measurements were done with trimeric Env proteins as analytes in solution and bivalent ligands (IgG-CD4 and VRC01 IgG) immobilized on a biosensor. The K_D, k_{on} and k_{off} are tabulated below the Octet sensorgrams.

FIG S5

Yield and Design Elements of NFL TD CC+ Trimers

HIV Env Seq ID	TD (BG505 Trimer Derived)														
	47	49	65	106	164	165	172	308	429	432	500	543	553	588	662
BG505	D	E	K	T	E	L	V	R	R	Q	R	N	S	R	A
JRFL	E-D	T-E	V-K	E-T	S-E	I-L	E-V	H-R	E-R	K-Q	K-R	L-N	N-S	G-R	E-A
SC422	E-D	K-E	K	E-T	A-E	I-L	T-V	T-R	E-R	K-Q	K-R	L-N	N-S	R	E-A
WITO	E-D	N-E	V-K	E-T	V-E	I-L	E-V	N-R	E-R	K-Q	R	L-N	S	K-R	E-A
16055	E-D	K-E	V-K	E-T	E	I-L	V	R	E-R	R-Q	A-R	Q	S	K	A
001428	E-D	R-E	V-K	E-T	E	I-L	A-V	R	E-R	R-Q	R	Q	S	K	A
ZM197M	E-D	K-E	V-K	E-T	E	V-L	Q-V	R	E-R	R-Q	S-R	Q	S	K	A
1086c	E-D	K-E	V-K	E-T	E	L	V	R	E-R	R-Q	E-R	Q	S	K	A

HIV Env Seq ID	CC		G			V3-FP			Added Glycans	~YIELD (mg) per Liter	Clade
	201	433	568	569	636	302	519	520			
BG505	I-C	A-C	L	T	S	N-Y	F-R	L-R	N332	2	A
JRFL	I-C	A-C	L	T-G	D	N-Y	F-R	L-R		4.5	
SC422	I-C	A-C	L	T-G	N	N-Y	F-R	L-R		4	B
WITO	I-C	A-C	L	T-G	D-G	N-Y	F-R	L-R		3	
16055	I-C	A-C	L	T-G	S-G	N-Y	F-R	L-R	N332	2.5	
001428	I-C	A-C	L-G	T	S-G	N-Y	F-R	L-R		2	C
ZM197M	I-C	A-C	L-G	T	D-G	N-Y	I-R	L-R	N160 N295 N332	2	
1086c	I-C	A-C	L-G	T	N-G	N-Y	F-R	L-R	N160	2	

Figure S5. Design elements of the NFL TD CC+ soluble trimers and yields. Related to Figure 6. Key elements of the NFL TD CC+ trimer design: the TD (or BG505 "Trimer Derived") residues, the gp41 glycine substitutions that promote trimer formation, the engineered disulfide CC (I201C-A433C), and the V3-FP (N302Y, F519R and L520R) substitutions that contribute to an increase in soluble trimeric NFL Env stability.

FIG S6

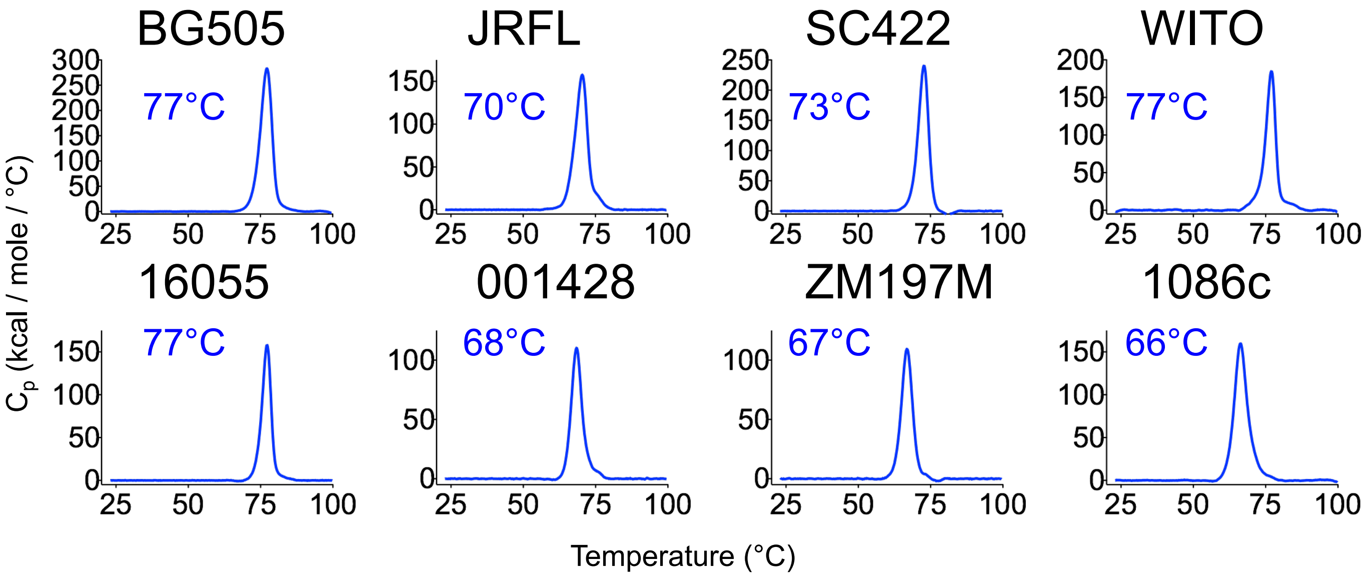

Figure S6. Stability of trimeric NFL proteins that include TD, CC, Glycines and V3-FP design elements. Related to Figure 6. DSC measurement of thermal transition midpoints of the NFL TD CC+ trimers following lectin and SEC purification.

Table S1.

NFL TD CC+	mAb	K_D (nM)	k_{on} (1/Ms) × 10^4	k_{off} (1/s) × 10^{-3}	HIV-1 Neutralization ug/mL
BG505	PGDM1400	**20**	6.5	1.3	0.001
	PGT145	**22**	9.4	2.1	0.025
	PG16	**45**	7.1	3.2	0.005
	VRC26	**14**	8.2	1.1	0.001
JRFL	PGDM1400	**15**	3.2	0.5	*
	PGT145	**3**	7.2	0.2	0.064
	PG16	**40**	7.3	2.9	0.023
	VRC26	**107**	12.1	12.9	>50
WITO	PGDM1400	**13**	3.5	0.5	0.001
	PGT145	**2**	8.0	0.2	0.0004
	PG16	**62**	3.8	2.3	0.0005
	VRC26	**108**	2.7	2.9	>50
SC422	PGDM1400	**53**	2.0	1.1	0.550
	PGT145	**24**	2.3	0.5	0.0004
	PG16	**156**	1.8	2.9	3.18
	VRC26	**205**	7.4	15	>50
16055	PGDM1400	**15**	7.6	1.2	0.001
	PGT145	**18**	13	2.3	0.008
	PG16	**17**	4.5	0.8	0.002
	VRC26	**8**	5.9	0.5	0.004
1086c	PGDM1400	**40**	2.6	1.0	*
	PGT145	**41**	4.1	1.7	*
	PG16	**55**	2.9	1.6	*
	VRC26	**78**	3.7	2.9	*
001428	PGDM1400	**32**	6.1	1.9	0.001
	PGT145	**26**	12	3.2	0.0006
	PG16	**44**	5.1	2.2	0.023
	VRC26	**43**	7.3	3.1	0.010
ZM197M	PGDM1400	**29**	2.1	0.6	0.029
	PGT145	**67**	2.2	1.5	0.523
	PG16	**124**	2.2	2.7	6.67
	VRC26	**18**	2.0	0.3	0.002

* Not determined

Table S1. Kinetic parameters of the NFL trimers with trimer-preferred V2-apex bNAbs and HIV-1 neutralization. Related to figure 6. The above table displays the affinity parameters measured by bio-layer light interferometry of the NFL trimers with trimer-preferred V2-apex bNAbs. Last column (right) shows the bNAb capacities to neutralize the HIV-1 strains from which the NFL trimers were derived as IC50 (ug/mL) values.
