# Supplementary material for: Glycine Substitution at Helix-to-Coil Transitions Facilitates the Structural Determination of a Stabilized Subtype C HIV Envelope Glycoprotein
Source: Immunity. 2017 May 16;46(5):792–803.e3. doi: 10.1016/j.immuni.2017.04.014 (PMC5439057; doi:10.1016/j.immuni.2017.04.014)
Supplement: Document S2. Article plus Supplemental Information [file mmc2.pdf]

Glycine Substitution at Helix-to-Coil Transitions Facilitates the Structural Determination of a Stabilized Subtype C HIV Envelope Glycoprotein

Graphical Abstract

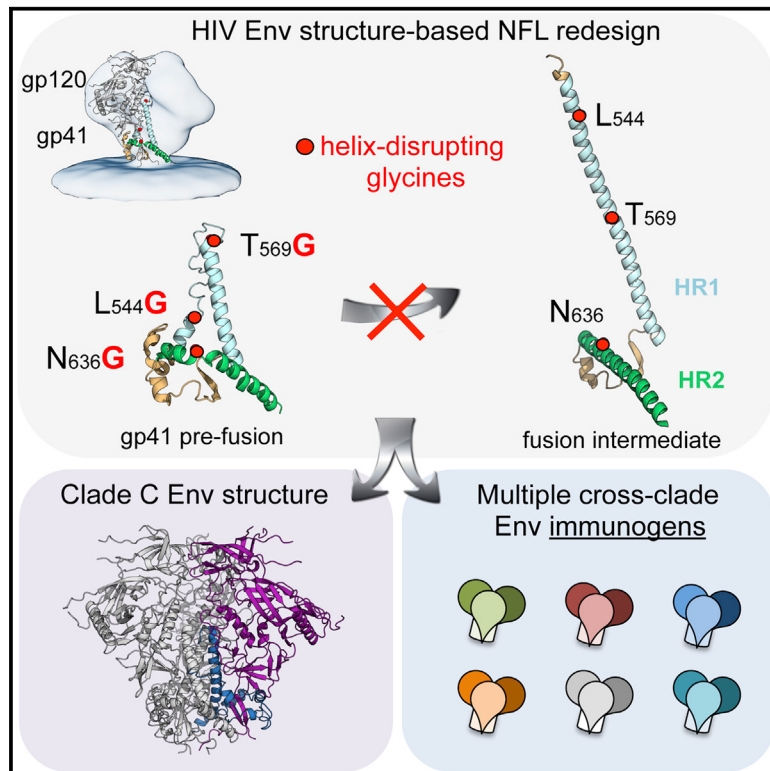

Authors

Javier Guenaga, Fernando Garces, Natalia de Val, ..., Andrew B. Ward, Ian A. Wilson, Richard T. Wyatt

Correspondence

wilson@scripps.edu (I.A.W.),
wyatt@scripps.edu (R.T.W.)

In Brief

The majority of HIV-1 infections worldwide emanate from subtype C strains. Guenaga et al. describe the 3.9 Å crystal structure of a stabilized subtype C native, flexibly linked (NFL) Env and multiple structure-guided design strategies that permit the generation of Env immunogens from diverse HIV strains.

Highlights

- Structure-guided design generates an HIV clade C Env crystal structure at 3.9 Å
- Cross-clade Env comparison discloses overall structural and N-glycan conservation
- The NFL structure reveals stabilizing TD contacts and the 201C-A433C disulfide CC
- NFL Env redesign permits the generation of immunogens derived from clades A, B, and C

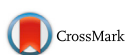

Glycine Substitution at Helix-to-Coil Transitions Facilitates the Structural Determination of a Stabilized Subtype C HIV Envelope Glycoprotein

Javier Guenaga,^{1,6} Fernando Garces,^{2,6} Natalia de Val,² Robyn L. Stanfield,^{1,2} Viktoriya Dubrovskaya,³ Brett Higgins,³ Barbara Carrette,¹ Andrew B. Ward,^{1,2,4} Ian A. Wilson,^{1,2,4,5,*} and Richard T. Wyatt^{1,3,4,7,*}

¹IAVI Neutralizing Antibody Center at The Scripps Research Institute, La Jolla, CA 92037, USA

²Department of Integrative Structural and Computational Biology, The Scripps Research Institute, La Jolla, CA 92037, USA

³Department of Immunology and Microbiology, The Scripps Research Institute, La Jolla, CA 92037, USA

⁴Scripps Center for HIV/AIDS Vaccine Immunology & Immunogen Discovery (CHAVI-ID), La Jolla, CA 92037, USA

⁵The Skaggs Institute for Chemical Biology, The Scripps Research Institute, La Jolla, CA 92037, USA

⁶These authors contributed equally

⁷Lead Contact

*Correspondence: wilson@scripps.edu (I.A.W.), wyatt@scripps.edu (R.T.W.)

<http://dx.doi.org/10.1016/j.immuni.2017.04.014>

SUMMARY

Advances in HIV-1 envelope glycoprotein (Env) design generate native-like trimers and high-resolution clade A, B, and G structures and elicit neutralizing antibodies. However, a high-resolution clade C structure is critical, as this subtype accounts for the majority of HIV infections worldwide, but well-ordered clade C Env trimers are more challenging to produce due to their instability. Based on targeted glycine substitutions in the Env fusion machinery, we defined a general approach that disfavors helical transitions leading to post-fusion conformations, thereby favoring the pre-fusion state. We generated a stabilized, soluble clade C Env (16055 NFL) and determined its crystal structure at 3.9 Å. Its overall conformation is similar to SOSIP.664 and native Env trimers but includes a covalent linker between gp120 and gp41, an engineered 201-433 disulfide bond, and density corresponding to 22 N-glycans. Env-structure-guided design strategies resulted in multiple homogeneous cross-clade immunogens with the potential to advance HIV vaccine development.

INTRODUCTION

A fundamental obstacle faced by all enveloped viruses is how to facilitate entry of their genetic material into susceptible host cells across two lipid bilayers. Most enveloped viruses converge upon a post-fusion “six-helix bundle” conformation of their envelope glycoproteins (Env) to accomplish fusion of the viral and host lipid bilayers, thereby facilitating entry of their genetic material into susceptible target cells (reviewed in [Colman and Lawrence \[2003\]](#)). Metastability is a requisite of envelope glycoproteins in terms of folding and assembly of a structure that can rearrange

at the appropriate time to attain its fusion-active form. Extended helical transitional intermediates are generated for viral fusion proteins after receptor and co-receptor engagement, either at the cell surface or at the lower pH of the endosome, propelling the fusion peptide to the opposite end of the trimer for insertion into the target cell membrane (reviewed in [Eckert and Kim, 2001](#)). Subsequently, the transitional intermediate collapses into a six-helix bundle, juxtaposing the viral and host cell membranes, allowing formation of a fusion pore that enables entry of the viral genetic material into the target cell.

For HIV, the Env is the sole neutralization target for antibodies on the viral surface and thus is of major interest for vaccine design. For decades, the generation of soluble mimics of Env was challenging due to its inherent metastability, in part because of the non-covalent association of the Env subunits, gp120 and gp41, due to furin cleavage of the precursor gp160 during natural infection to attain its fusion-active conformation ([Berger et al., 1991](#); [Moore et al., 1990](#)). An engineered disulfide linking the two subunits and a key mutation, I559P, in heptad repeat 1 (HR1) resulted in the first native-like Env soluble mimic, the SOSIP trimer ([Binley et al., 2000](#); [Sanders et al., 2002](#)). However, it was not until the generation of the subtype-A-derived BG505 SOSIP.664, with accompanying antigenicity, stability, and high-resolution structural data, that these trimers became widely considered and utilized as faithful mimics of the HIV spike ([Julien et al., 2013](#); [Lyumkis et al., 2013](#); [Sanders et al., 2013](#)). SOSIP trimers have been derived from many other Env sequences but can result in mixtures of ordered and disordered oligomers that can be “rescued” either by negative or positive selection ([Guenaga et al., 2015a](#); [Julien et al., 2015](#); [Pugach et al., 2015](#)). The initial cryoelectron microscopy (cryo-EM) and crystal structures of clade A BG505 SOSIP.664 ([Julien et al., 2013](#); [Lyumkis et al., 2013](#); [Pancera et al., 2014](#)) have been followed by Env structures for subtypes B and G ([Lee et al., 2016](#); [Stewart-Jones et al., 2016](#)). However, high-resolution clade-C-derived Env structures, the subtype that constitutes the vast majority of HIV infections worldwide, have not yet been obtained. In the past year, we reported the engineering of an uncleaved soluble Env mimic, the native, flexibly linked (NFL) trimer. This design includes the I559P

mutation present in SOSIP trimers but uses an extended flexible linker to replace the furin cleavage site between the two Env subunits, rendering these trimers both covalently linked and cleavage independent (Sharma et al., 2015). The native-like NFL trimers display antigenic and biochemical characteristics comparable to SOSIP trimers but do not require cleavage of the two Env subunits by cellular or exogenous furins (Guenaga et al., 2015b; Sharma et al., 2015). Like the SOSIP, the NFL design works best on a subset of Env sequences and, in its original design, is particularly inefficient at generating high yields of trimers derived from clade C strains.

We engineered a soluble trimer based on an Indian subtype C HIV Env sequence, called 16055 NFL “TD,” where TD refers to the reversion of eight BG505–“trimer-derived” residues that substantially improve the propensity to form native-like trimers (Guenaga et al., 2015b). To improve further on the TD design, we introduced targeted glycine substitutions in gp41 at helix-to-coil transitions to disfavor the post-fusion state of Env to generate highly homogeneous, soluble NFL clade C trimers. Aided by these design advances, we derived the crystal structure of the disulfide-stabilized (I201C-A433C) 16055 NFL TD CC (T569G) trimer in complex with the broadly neutralizing antibodies (bNAbs), PGT124 and 35O22, at 3.9 Å resolution, providing a high-resolution Env structure derived from a subtype C primary isolate. Comparison of this new structure to the available subtype A, B, and G structures reveals overall similarities, as well as interesting strain-dependent differences. In the NFL trimer structure, electron density is present for at least one sugar moiety of the 22 N-glycans visible from the 29 potential N-glycosylation sites (PNGS) (n.b., the others are mainly in disordered loops). The complex structure also defines the details of PGT124 bAb interactions with Env in the context of a well-ordered trimer. Aided by the structural analysis, additional trimer redesign integrating TD, CC, glycine targeting, and further structure-based single-amino-acid modifications resulted in the generation of highly stable and homogeneous NFL soluble Env mimics for multiple subtypes. These improved designs advance our understanding of HIV Env structural interactions and our capacity to generate well-ordered NFL Env trimers from different clinical isolates derived from multiple subtypes. These advances lay a foundation for further structure-guided modifications to improve trimer conformational homogeneity, stability, and yield, providing an expanded repertoire of clade C and other HIV vaccine candidate immunogens. In a related accompanying paper, the immunogenic properties of well-ordered 16055 NFL TD CC trimers were evaluated in non-human primates (Martinez-Murillo et al., 2017).

RESULTS

Glycine Substitutions in gp41 Promote Soluble Env Trimer Formation

To generate clade C NFL Env glycoproteins amenable to crystallization, we pursued a strategy to promote well-ordered trimer formation. To disfavor the Env post-fusion state and consequently promote pre-fusion trimer formation, we introduced glycine substitutions into key coil-to-helix transitional areas of the gp41 subunit, guided by the pre-fusion high-resolution Env trimer structures (Figure 1A) (Garces et al., 2015; Gristick et al.,

2016; Julien et al., 2013; Lyumkis et al., 2013; Pancera et al., 2014). Putative helical intermediates of Env are formed following receptor-coreceptor engagement, liberating gp41 from gp120-imposed conformational constraints. Because glycines disfavor helix formation, we substituted G residues at selected coil-to-helix junctions to inhibit conformational transitions to putative intermediate and structurally defined post-fusion, helix-dominated conformations. To test the glycine substitution strategy, we first generated NFL trimeric proteins based on the transmitted/founder subtype C South African HIV Env sequence, 1086c, introducing TD substitutions previously described (Guenaga et al., 2015b). TD stabilization alone was insufficient to generate native-like trimers for 1086c as determined by both SEC and EM (Figure 1B and Figure S1A), in contrast with their previous substantial impact on trimer formation for Indian clade C 16055 NFL and clade B JRFL NFL (Guenaga et al., 2015b).

To design more homogeneous 1086c NFL trimers and potentially for a wide range of trimers from multiple strains, we identified inferred coil-to-helix transitional regions in gp41 guided by the high-resolution structure of the pre-fusion BG505 SOSIP.664 and gp41 post-fusion structures (Buzon et al., 2010; Garces et al., 2015; Gristick et al., 2016; Julien et al., 2013; Liu et al., 2009; Lyumkis et al., 2013; Pancera et al., 2014). The pre-fusion gp41 regions of interest were located in the N-terminal HR1 loop (HR1_N), which connects the central HR1 helix to the fusion peptide proximal region (FPPR), and in the region proximal to the N-glycan at residue 637 in HR2, where the HR2 helices bend to wrap around the gp120 N and C termini (Figure 1A). Crystal structures reveal that these coiled regions in gp41 transition to helices during the pre-fusion to the post-fusion state (Buzon et al., 2010; Shu et al., 2000; Tan et al., 1997). Therefore, within the context of the 1086c NFL TD protein that displays no significant amount of ordered trimers, we generated selected glycine substitutions to test their effect on ordered trimer formation. Residues T536, L537, L544, L568, T569, N636, and Y638 were individually substituted with glycines and evaluated by size exclusion chromatography (SEC), EM, and immunoprecipitation (IP). Following initial NFL glycoprotein purification by lectin-affinity chromatography, SEC profiles revealed a marked change in the proportions of monomeric, dimeric, and aggregated 1086c glycoproteins carrying the selected glycine substitutions (Figure 1B). 1086c NFL TD glycoproteins with glycine substitutions at positions 568 and 569 displayed the most improved trimer formation relative to the TD variant, with reduced monomer, dimer, and higher-order forms (Figure 1B). Substitutions at 544 and 636 showed moderate improvement, whereas substitutions at 536, 537, and 638 showed no improvement (Figure 1B).

EM negative-stain analysis of the trimeric proteins showed a marked increase in native-like trimers for variants with glycine substitutions at residues 568, 569, and 636 and moderately for 544, generally consistent with the SEC analysis (Figure S1A). IP of NFL TD protein supernatants using the CD4 binding site (CD4bs), trimer-preferring broadly neutralizing antibody (bNAb) VRC03, and the trimer-independent CD4bs bNAb, VRC01, yielded results consistent with the EM analysis. For example, stronger intensity VRC03 IP bands were detected for NFL TD variants with glycine substitutions at residues 568 and 569 compared to isogenic trimers lacking these changes (Figure S1B). We also evaluated various single-, double-, and

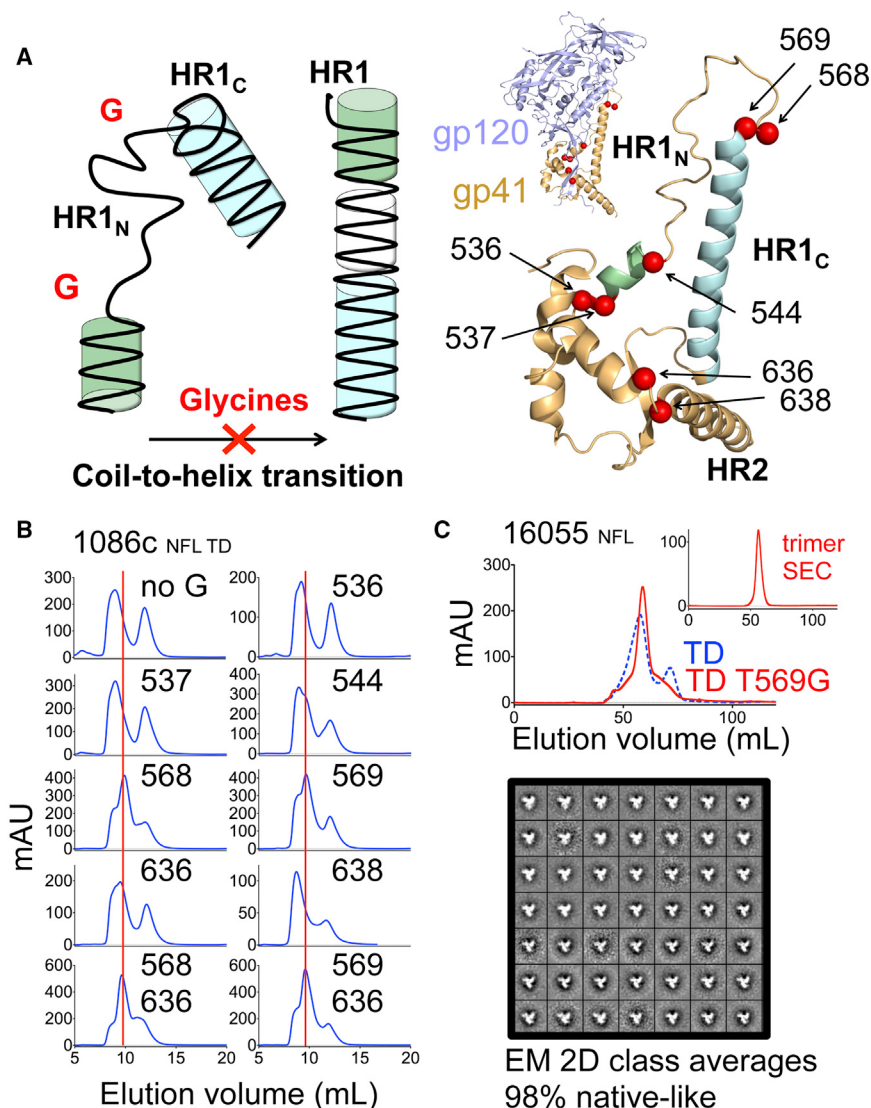

Figure 1. Env gp41 Glycine Substitutions Increase Native-like Trimer Formation

(A) Schematic model of the targeted glycine strategy to attenuate helical secondary structural transitions to the post-fusion form (left) and location of the tested glycine substitutions (red spheres) displayed on the crystal structure of the gp41 subunit in BG505 SOSIP.664 (PDB: 5CEZ) (right).

(B) SEC profiles of subtype C 1086c NFL TD variants with glycine substitutions at the indicated residues and TD as a control with no glycines. The vertical dotted red line denotes the elution volume of native-like, well-ordered trimers.

(C) SEC profiles of HIV-1 subtype C 16055 NFL TD trimers with and without T569G after lectin chromatography purification. Shown in the upper right is the SEC profile corresponding to a re-run of the trimer fractions. 2D class averages from negative-stain EM of the trimer fraction is shown below. See also Figures S1 and S2.

16055 NFL TD (T569G) trimers displayed a native-like conformation as determined by negative-stain EM (Figure 1C).

These results revealed that helix-disrupting glycine substitutions at critical helix-to-coil interfaces disfavor transitions toward the receptor-triggered putative HIV Env transitional intermediate and, specifically, that glycine substitutions at the HR1_N residues 568 and 569 and HR2 residue 636 increased native-like Env trimer formation.

The Clade C Cleavage-Independent 16055 NFL TD CC T569G Trimer Shares Structural Homology with Clade A, B, and G Envs

Several HIV-1 subtype C Env-derived SOSIP and NFL trimers are well described

biochemically and by low-resolution EM (Guenaga et al., 2015a, 2015b; Julien et al., 2015; Ringe et al., 2015; Sharma et al., 2015). However, no high-resolution structure of a soluble subtype C Env trimer has been determined. We therefore used the highly homogeneous T569G variant of 16055 NFL TD CC trimer to generate crystals that diffracted to 3.9 Å in complex with bNAbs PGT124 and 35O22 (Garces et al., 2014; Huang et al., 2014; Pancera et al., 2014). From this ternary complex, we were able to determine the crystal structure of a clade C Env NFL trimer by molecular replacement (see STAR Methods, Figure 2A, and Table 1).

Overall, the structure of 16055 NFL TD CC (T569G) in complex with PGT124 and 35O22 closely resembles those of soluble subtype A BG505 SOSIP.664, subtype G X1193.c1 SOSIP.664, and full-length native subtype B JRFL Env ΔCT Env trimers (Figure 2B) (Garces et al., 2015; Julien et al., 2013; Lee et al., 2016; Pancera et al., 2014; Stewart-Jones et al., 2016). The similarity strengthens the interpretation that the engineered soluble NFL trimers, like SOSIP, are close mimics of HIV Env. The largest

triple-glycine combinations and concluded that single-L568G or -T569G and double-L568G-N636G or -T569G-N636G substitutions markedly improved the generation of well-ordered 1086c NFL TD trimers. Based on the VRC03 IP band intensity, triple combinations (T569G-L544G-N636G and L568G-L544G-N636G) did not appear to be better than the double substitutions and were not pursued further in this study (Figure S1B).

To assess the transferability of this strategy to other HIV Env sequences, we introduced the 1086c-effective T569G substitution into the JRFL NFL TD and 16055 NFL TD glycoproteins (Guenaga et al., 2015b) and assessed its effect on trimer homogeneity by SEC and differential scanning calorimetry (DSC). The T569G substitution greatly increased trimer formation and homogeneity in both clade B and C NFL TD contexts (Figures 1C, S1C, S2A, and S2B). The L568G mutation conferred similar results in the context of 16055 NFL TD as assessed by SEC and DSC, displaying comparable levels of homogeneity and stability (only 0.4°C less) (Figure S1C). In fact, with no additional purification except for isolation of the trimer fraction from the SEC, 98% of the

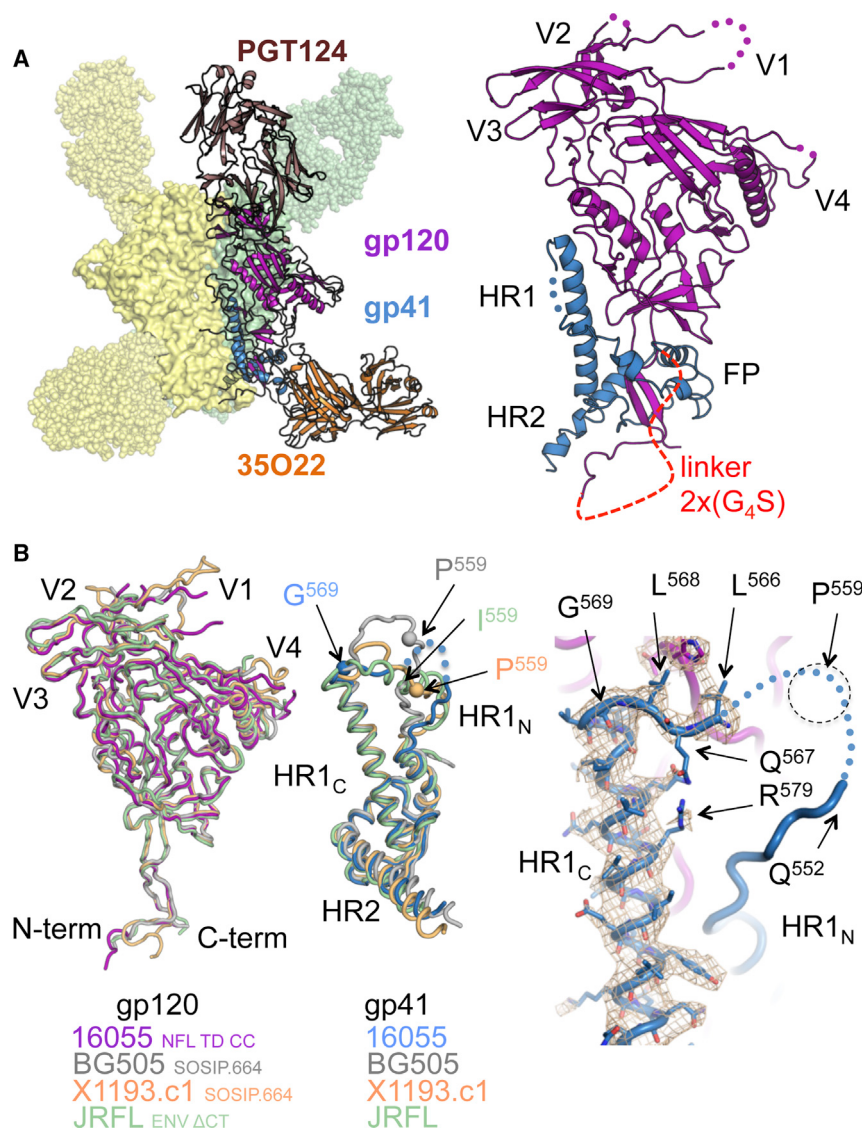

Figure 2. The Cleavage-Independent Subtype C HIV-1 Env 16055 NFL TD CC Trimer Shares Homology with Other Env Trimer Structures

(A) Crystal structure at 3.9 Å resolution of the 16055 NFL TD CC (T569G) trimer (gp120 purple, gp41 blue, PDB: 5UM8) in complex with Fabs PGT124 (brown) and 35O22 (orange).

(B) Superimposition of the gp120 subunits of soluble Env trimers derived from HIV-1 clade C NFL (magenta), clade A BG505 SOSIP.664 (gray, PDB: 5CEZ), clade G X1193.c1 SOSIP.664 (light orange, PDB: 5FYJ), and clade B JRFL Env ΔCT (green, PDB: 5FUU) (left). Corresponding overlap of the gp41 subunits of 16055 (blue), BG505 (gray), X1193.c1 (light orange), and JRFL Env (green) (middle). Close-up view of the HR1 region of the NFL trimer showing the T569G mutation and residues downstream that demarcate the region that undergoes the coil-to-helix transition in the post-fusion structure (right).

See also Figures S2 and S3.

differences among Env structures were localized to variable regions V1, V2, V4, and V5 of gp120 and the HR1_N region spanning residues 545–569 of gp41 (Figure S2C). With the exception of the V3, variable regions of gp120 differ both in length and conformation and contribute to antigenic variation with respect to antibody recognition (Figure S2C). The observed HR1_N differences may be relevant to trimer stability and, perhaps, to the capacity of different Env sequences to form stable, soluble trimers, since the well-documented I559P substitution (Sanders et al., 2002) and the T569G described here are located in this flexible region, and both affect trimerization propensity (Figure S2C). Another difference in these NFL trimers is the flexible (G₄S)₂ linker connecting gp120 to gp41 that eliminates the need for furin cleavage (Sharma et al., 2015), for which only the first three glycine residues are visible in the NFL structure (Figure 2A).

Structural alignment between the BG505 SOSIP.664 and the 16055 NFL TD CC (T569G) structures revealed low C_α RMSD values for the gp140 protomer (0.7 Å) and the respective gp120 (0.6 Å) or gp41 (0.6 Å) subunits (Figure S3A). Although res-

idues 553–565 of the gp41 HR1_N were not resolved in the 16055 structure, L566, Q567, L568, and G569 downstream of the I559P substitution were interpretable and offer some insights into how the 569 glycine substitution contributed to the generation of stable clade C Env NFL trimers (Figure 2B). The orientation and location of these four residues (566–569) in 16055 NFL TD CC (T569G) deviate from that in the BG505 SOSIP.664 and X1193.c1 SOSIP structures (PDB: 5CEZ and PDB: 5FYJ) but follow an initial path more similar to that in the clade B JRFL Env ΔCT (PDB: 5FUU), although different in conformation (Figures 2B and S2C). The helix-disrupting capacity of the T569G mutation disfavors the extension of the HR1_C helix, helping HR1_N remain in its flexible coil form observed in the SOSIP structures (Figures 2B and S2C). The increase in NFL trimer formation may also result from generation of new local stabilizing contacts in the HR1_N region; however, no increase in the overall trimer thermal transition midpoint (T_m) was detected (Figure S2A). Perhaps the glycine substitution simply decreases the tendency of this region to adopt a transitional intermediate form of gp41, therefore favoring the pre-fusion state.

These analyses demonstrated that the crystal structure of the furin-independent clade C Env 16055 NFL TD CC (T569G) shares overall structural homology with other Envs, revealing differences in the surface-exposed variable regions and in the more occluded trimer-axis proximal region of gp41 HR1_N.

Env N-Glycosylation Differences between Strains Identify Potential Sites of Vulnerability for Antibody Recognition

HIV has evolved immune evasion mechanisms that pose a major challenge to vaccine design. Env surface N-glycosylation is

Table 1. Data Collection and Refinement Statistics

Data collection	16055 NFL TD CC (T569G) + PGT124 + 35O22
Beamline	APS-23ID-D
Wavelength (Å)	1.03320
Space group	P6 ₃
Unit cell parameters (Å)	a = b = 126.5, c = 314.1
Resolution (Å)	50.0-3.94 (4.02-3.94) ^a
Observations	125,205
Unique reflections	25,153 (1,224)
Redundancy	5.0 (4.9)
Completeness (%)	100 (100)
$\langle I \rangle / \langle \sigma_I \rangle$	7.5 (1.1)
CC _{1/2} (%) ^b	92.3 (64.1)
R _{sym} (%) ^c	19.4 (150.5)
R _{pim} (%) ^d	9.6 (75.0)
Refinement statistics	
Resolution (Å)	49.25-3.94
Reflections (work)	23,799
Reflections (test)	1,253
R _{cryst} (%) ^e	27.4 (37.7)
R _{free} (%) ^f	31.8 (39.1)
Average B-value (Å ²)	
All protein atoms	156
gp120	126
gp41	149
PGT124 (V _H /V _L)	140
PGT124 (C _L /C _H 1)	150
35O22 (V _H /V _L)	163
35O22 (C _L /C _H 1)	242
Glycans	155
Wilson B-value (Å ²)	119
RMSD from ideal geometry	
Bond length (Å)	0.002
Bond angles (°)	0.58
Clashscore ^g	6.6
Ramachandran statistics (%)	
Favored	90.6
Outliers	1.8
PDB ID	PDB: 5UM8

^aNumbers in parentheses refer to the highest resolution shell.

^bCC_{1/2} = Pearson Correlation Coefficient between two random half datasets

^cR_{sym} = $\sum_{hkl} \sum_i |I_{hkl,i} - \langle I_{hkl} \rangle| / \sum_{hkl} \sum_i I_{hkl,i}$, where $I_{hkl,i}$ is the scaled intensity of the ith measurement of reflection h, k, l , $\langle I_{hkl} \rangle$ is the average intensity for that reflection, and n is the redundancy (Weiss and Hilgenfeld, 1997).

^dR_{pim} is a redundancy-independent measure of the quality of intensity measurements. $R_{pim} = \sum_{hkl} (1/(n-1))^{1/2} \sum_i |I_{hkl,i} - \langle I_{hkl} \rangle| / \sum_{hkl} \sum_i I_{hkl,i}$, where $I_{hkl,i}$ is the scaled intensity of the ith measurement of reflection h, k, l , $\langle I_{hkl} \rangle$ is the average intensity for that reflection, and n is the redundancy.

^eR_{cryst} = $\sum_{hkl} |F_o - F_c| / \sum_{hkl} |F_o| \times 100$

^fR_{free} was calculated as for R_{cryst}, but on a test set comprising 5% of the data excluded from refinement.

^gNumber of unfavorable all-atom steric overlaps ≥ 0.4 Å per 1,000 atoms.

an important evasion mechanism in which self-glycans occlude the underlying Env protein surface. With an average of 80–90 N-linked glycans per Env trimer, this protective coverage is often referred to as the “glycan shield” (Lasky et al., 1986; Wei et al., 2003). The 16055 NFL TD CC trimers were produced in a GnT1^{−/−} mutant cell line that results in processing of N-glycans to Man_{5–9}GlcNAc₂ oligomannose forms. The ternary complex of PGT124:16055 NFL TD CC:35O22 was deglycosylated by Endoglycosidase H (Endo H) treatment to facilitate crystallization. At least one sugar moiety for 22 glycans was visible in the electron density maps out of the 29 potential N-linked glycosylation sites (PNGS) in the 16055 Env sequence. For 15 N-glycans out of the 22 visible in the structure, we observed density beyond the peptide-proximal N-acetylglucosamine moiety, indicating protection from Endo H cleavage. The other glycan positions for which we did not observe interpretable density were mainly in disordered regions or loops. In comparison, 17 glycans had observable electron density in the 27 PNGS of the 3 Å structure of BG505 SOSIP.664 (Figure 3) (Garces et al., 2015). The V1 and C2 regions of 16055 were more heavily glycosylated, possessing five additional PNGSs at N138, N147, N230, N241, and N289. In contrast, the C3 region of BG505 had two additional potential glycosylation sites at N339 and N355 (Figure 3). Breaches in the glycan shield, such as those corresponding to N230–N241–N289 and N339–N355 for BG505 and 16055, respectively, may define strain-specific sites of vulnerability as recently shown for Env vaccine-elicited antibodies in rabbits (Crooks et al., 2015; Klasse et al., 2016; McCoy et al., 2016). Some of these rabbit antibodies neutralize the HIV BG505 strain by exploiting a gap in the glycan shield at the N241 region, thereby more readily accessing the exposed protein surface. However, the majority of HIV strains are glycosylated at N241, as is 16055, or possess a different protein sequence in this region, making them resistant to neutralization by such antibodies (Figure 4A).

Almost all bNAbs isolated from infected patients, such as PGT124 that targets the 332 N-glycan and V3 base, incorporate glycans as part of their epitope, contacting a combination of sugar moieties and underlying protein residues (Figure 4B). In the 16055 crystal structure, PGT124 penetrates into a cleft surrounded by glycans N156, N301, and N332 (Figure 4B). The antibody mostly avoids the N-glycans at residues N156 and N301 but makes contact with N332 and the conserved V3³²⁴G(N/D)IR³²⁷ motif as previously demonstrated for PGT124 in the context of a gp120 core (Garces et al., 2014). The NFL trimer structure shows additional van der Waals interactions between PGT124 CDRL3 (Ser93, Ser95), CDRH3 (Phe100k), and gp120 V1 (Thr137) and backbone contacts between CDRL3 (Arg94) and the base of gp120 V3 (Ile322) (Figure 4B). Phe100g at the tip of CDRH3 that is important for the neutralization activity of PGT124 in a paratope scan (Garces et al., 2014) interacts with Env Gln328, Tyr330, Thr415, and Pro417 (Figure 4B).

These data were consistent with the limited neutralizing capacity of strain-specific antibodies that exploit gaps in the HIV Env N-glycan shield, while bNAbs evolve to accommodate glycan recognition as a means to more consistently penetrate the shield.

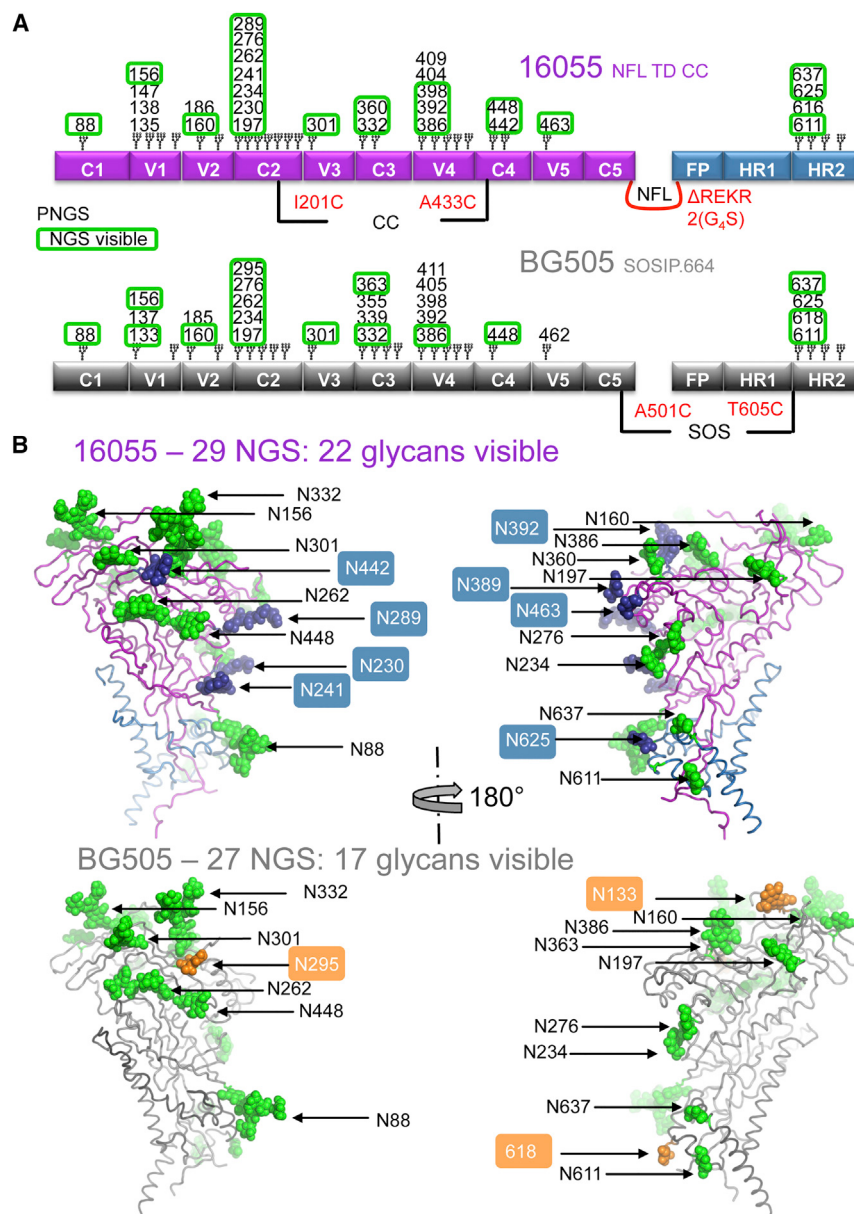

Figure 3. N-Glycosylation Sites in the 16055 NFL TD CC Structure Differ from Those in the BG505 SOSIP.664 Structure

(A) Schematic representation of the 16055 NFL and BG505 SOSIP trimer constructs, with all PNGS numbered on Env regions and those visible in either structure highlighted in green boxes. (B) 16055 NFL TD CC (T569G) structure (top) with gp120 (magenta), gp41 (blue), and BG505 SOSIP.664 structure (where N137 was deleted) (bottom, gray) (PDB: 5CEZ) in cartoon representation, with visible N-glycans as green or blue spheres. Blue spheres represent glycans visible in the 16055 NFL TD CC (T569G) structure that are either not ordered in the BG505 SOSIP.664 structure or are not PNGS in BG505 Env, and the orange spheres represent the reverse in BG505 SOSIP.664.

apex contacts Thr128 and Cys126 of the adjacent protomer, presumably strengthening apical trimer interactions. Arg429 and Gln432 contact Asn425, Thr202, and Leu116, creating a network of interactions that may stabilize the pre-bridging sheet (Figure S3B). TD Lys65 and Thr106 do not contribute to any obvious productive interactions.

The 16055 NFL TD CC (T569G) crystal structure illustrates formation of the I201C-A433C disulfide (CC) that was engineered to prevent CD4-induced trimer rearrangements and subsequent exposure of non-neutralizing epitopes (Figure 5A) (Guenaga et al., 2015b; Kwon et al., 2015). As the 16055 NFL TD CC (T569G) and BG505 SOSIP.664 trimer structures displayed similar conformations, with a C α RMSD of 0.6 Å for gp120 (Figure S3A), the engineered CC does not appear to significantly alter the conformation of the pre-CD4 form of the CD4bs. This region constitutes a conserved neutralizing determinant, so

maintenance of an unperturbed CD4bs is an important consideration for HIV vaccine design. Relevant to this latter point, affinity and avidity measurements for bNAb Fabs VRC01 and VRC06b to NFL TD trimers with and without the CC disulfide were of similar magnitude (Figure S4). For sCD4, although the overall K_D values were the same, CD4 had a slightly faster on rate but faster off rate with the NFL TD CC trimers.

Comparison of the gp120 subunit of the 16055 NFL TD CC (T569G) trimer structure with the CD4-liganded gp120 core (PDB: 3JWO) (Pancera et al., 2010) revealed differences between these two gp120 conformations, as previously described (Pancera et al., 2014) (Figures 5B and 5C). This comparison suggested a receptor-triggered model, whereby gp120 conformational changes culminate in the release of restraints on gp41. In this model, CD4 interaction with gp120 induces a displacement of the β 20 and β 21 loop, triggering Env rearrangements

The High-Resolution 16055 NFL Structure Reveals the Stabilizing Effects of the TD and CC Substitutions

The 16055 NFL trimer contains eight engineered TD residues, the CC disulfide, and the T569G glycine substitution. The NFL structure elucidated the role of these design elements in generating native-like trimers with increased stability (Guenaga et al., 2015b). We observed interactions for six of the eight TD residues located in four distinct areas of Env: gp120-gp41 interface (Arg500), gp120 N terminus (Asp47 and Glu49), pre-bridging sheet (Arg429 and Gln432), and apex domain (Leu165) (Figure S3B). The Arg500 substitution at the C terminus of gp120 contacts the adjacent gp41 His619 and gp120 N-terminal residues Gly32 and Leu34, strengthening the association of gp120 with gp41 at the base of the trimer. Asp47 and Glu49 in the N-terminal region of gp120 contact Lys487 and Asp99, respectively, anchoring this flexible region. Leu165 at the tip of the trimer V2

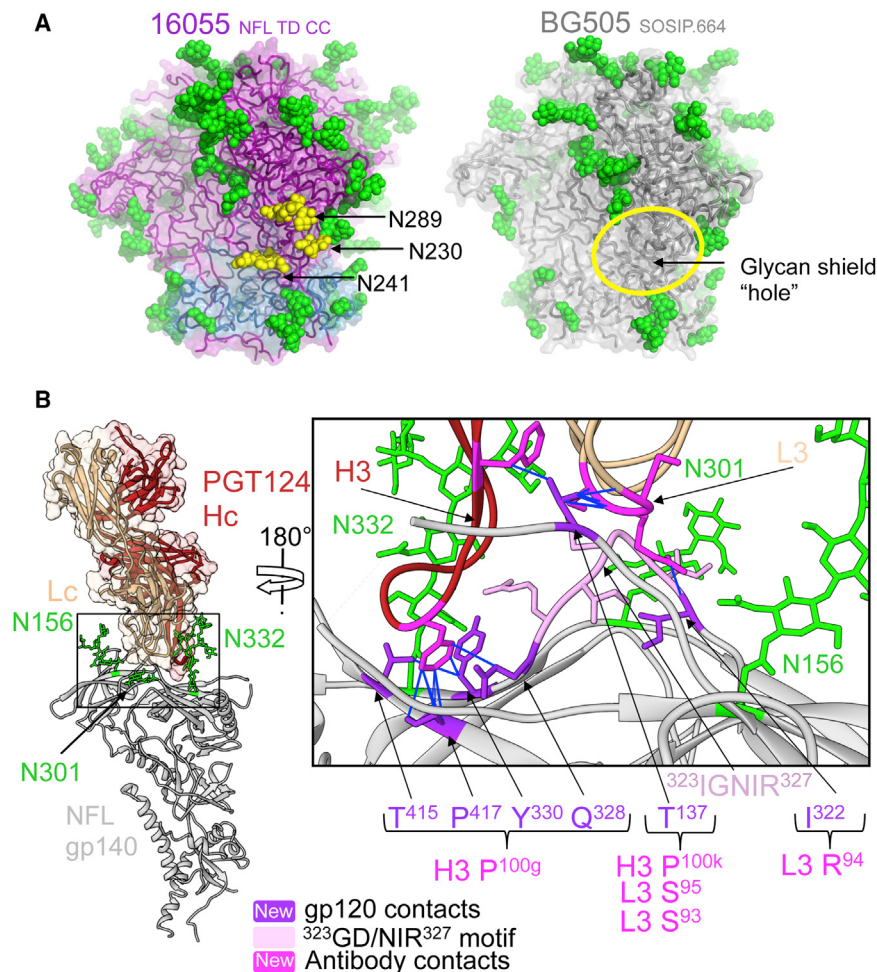

Figure 4. PGT124 Displays Broad Recognition of the Glycan Shield, while Autologous NABs Exploit Gaps in the Shield

(A) 16055 NFL TD CC (T569G) structure with gp120 (magenta), gp41 (blue) (left), and BG505 SOSIP.664 structure (gray, right) in cartoon and surface-mode representation, with glycans as green or yellow spheres. Yellow spheres denote glycans present on 16055 Env that are not present in BG505 Env that produce a glycan hole that is targeted by vaccine-elicited antibodies (Klasse et al., 2016; McCoy et al., 2016).

(B) 16055 NFL TD CC (T569G) structure in cartoon representation (gp140 gray), with Fab PGT124 (brown) in surface mode and proximal N-glycans N156, N301, and N332 in green (left). Close-up view of the PGT124-interacting region, where the antibody heavy and light chains (dark and light brown, respectively) are represented as a cartoon and the Env gp120 subunit in gray with glycans in green sticks. Previously described antibody contacts [IG(D/N)IR] with a gp120 core are colored pink (Garces et al., 2014), while new contacts in the context of trimeric Env are colored purple, with the corresponding interacting antibody residues colored in magenta.

that lead to co-receptor engagement. Serine 42 of CD4 would “push” gp120 glutamine 428 (β 20), facilitating release of β 21 contacts with β 3 (Figure 5B). The subsequent swap of β 3 and β 2 facilitates rearrangement of the variable domains (V1/V2), liberating the V3 region to engage with the co-receptor. However, the engineered CC disulfide specifically prevents dissociation of β 21 from β 3, acting as a molecular tether that locks gp120 into its pre-receptor conformation (Figure 5A). Other structural differences were observed that connect the CD4-bound trigger point (β 20- β 21) with release of gp41 HR1 from gp120. Helix α -1 in the CD4-liganded core was tilted a few degrees toward the adjacent α -0 (Figure 5C), suggesting that CD4 receptor and subsequent CCR5 coreceptor binding trigger a chain of events that lead to the formation of a putative intermediate Env structure.

In sum, these analyses demonstrated that the TD, CC, and T569G mutations combine to preserve the pre-fusion state of the NFL trimers by disfavoring conformational changes in gp120 and gp41 that otherwise would favor the fusion-active form.

Targeting Additional HIV Env Metastable Regions Facilitates Generating NFL Trimers Derived from Several Subtypes

Metastability is likely a requisite for Env to mediate receptor-triggered viral entry at neutral pH. Regions of Env, such as the var-

iable region 3 (V3) and the fusion peptide (FP), undergo large translational and conformational rearrangements following receptor-coreceptor engagement on target cells (Figure 6A) (Buzon et al., 2010; Luftig et al., 2006; Pancera et al., 2014). We sought to further stabilize the soluble NFL trimers beyond the stabilizing mutations described in the structure

presented here by further reducing the inherent Env metastability. We reasoned that conformational stabilization of these trimeric immunogens might be important for preservation and presentation of quaternary-dependent neutralizing epitopes following vaccination and affinity maturation while avoiding Env states that expose non-neutralizing epitopes. We selected JRFL NFL TD CC (T569G) as a test case because of its inherent lower thermostability in comparison with BG505 or 16055 native-like trimers (Guenaga et al., 2015a, 2015b; Sharma et al., 2015). Following a structure-guided approach, a number of V3 or FP residues were scanned for their capacity to increase inter- or intra-protomer interactions. We made substitutions of selected residues in these regions to large side chains—mainly, Trp, Tyr, and Arg—to fill pockets or make potential additional contacts to enhance subunit and trimer interactions. The initial scan identified three V3-FP stabilizing mutations (V3 N302Y and FP F519R and L520R) that resulted in increased trimer melting temperatures. DSC analysis of the triple mutant (N302Y F519R L520R) JRFL NFL TD CC (T569G) confirmed a 6°C increase in the T_m (Figure 6B). We introduced the V3-FP mutations into subtype A BG505 NFL CC and subtype C 16055 NFL TD CC (T569G) and determined that their respective T_m 's also increased by 6°C (Figure 6B). By applying all elements of the current design strategy (TD, CC, gp41

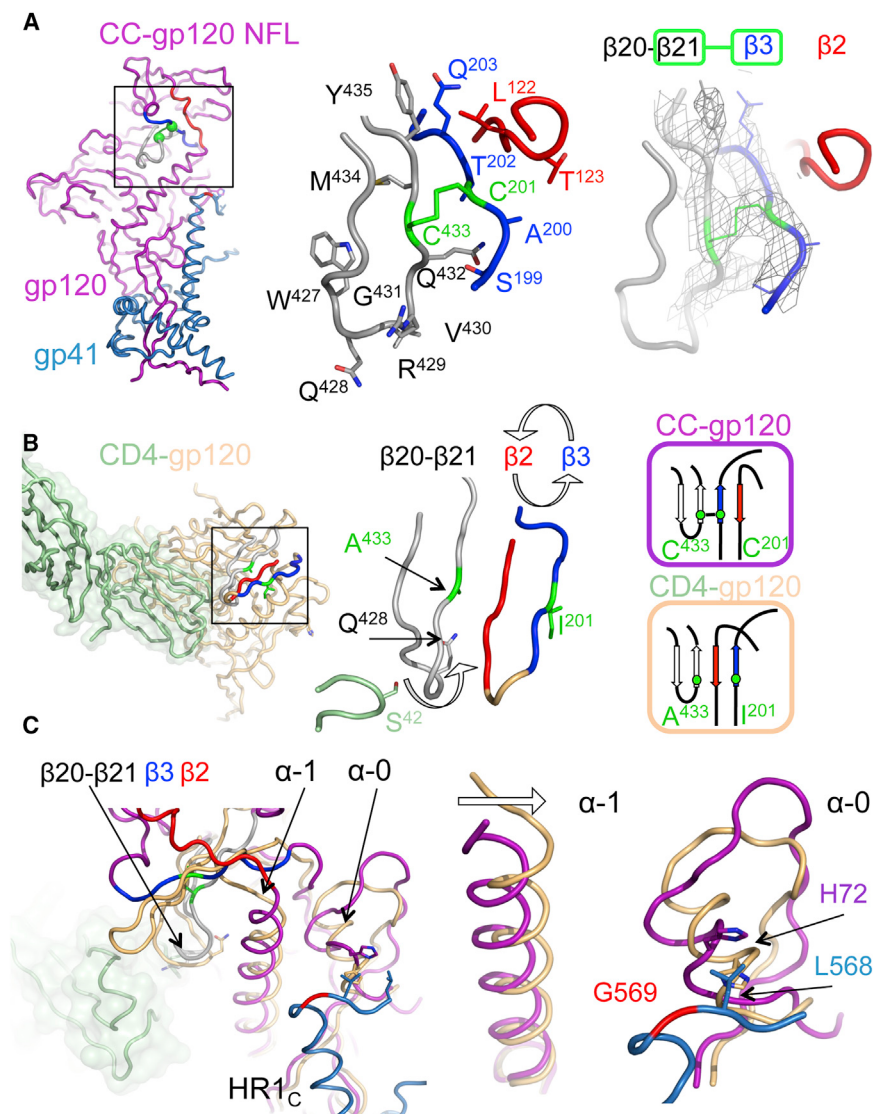

Figure 5. The Engineered Disulfide I201C-A433C Prevents CD4-Induced Env Rearrangements, Locking Env in the Pre-fusion State

(A) 16055 NFL TD CC (T569G) protomer (gp120, magenta; and gp41, blue) showing the location of the engineered disulfide (CC) in the pre-bridging sheet region ($\beta 20$ – $\beta 21$, gray), ($\beta 3$, blue), ($\beta 2$, red), and the C201–C433 disulfide (green) (left). Close-up view of the pre-bridging sheet region (middle) and the 2Fo-Fc electron density map contoured at 1.0σ of the gp120 subunit $\beta 21$ – $\beta 3$ region showing formation of the CC disulfide (right).

(B) CD4-ligated core gp120 (sCD4 in green and gp120 in light brown, PDB: 3JWO) with bridging sheet region colored the same as in (A) (left). Close-up view of the bridging sheet, illustrating the swap of $\beta 2$ and $\beta 3$ and the displacement of Q428 by CD4 (middle). Diagrams of the two states of the bridging sheet, as seen in the NFL and SOSIP structures (purple) and in the CD4-bound gp120 core (light brown) (right).

(C) Superimposition of the CD4-unliganded NFL structure with that of the CD4-ligated gp120 core. Some of the gp120 elements in the proximity of the CD4 binding site adopt different conformations between the pre-fusion state (purple, NFL Env structure) and the CD4-ligated state (brown, gp120 core structure).

See also Figure S4.

glycines, and V3-FP mutations, herein called NFL TD CC+) to other HIV Env sequences from clades B (SC422 and WITO) and C (1086c, 001428 and ZM197M), we sought to demonstrate the general applicability of our soluble NFL TD CC+ trimer platform (Figure S5). The NFL TD CC+ design made use of double glycine substitutions, either G568–G636 or G569–G636, as needed to increase homogeneity of the highly stabilized trimers. The resulting NFL TD CC+ trimers were purified via lectin affinity followed by SEC and then subjected to DSC analysis to assess their overall stability and homogeneity. SEC profiles revealed prominent trimer peaks at the expected elution volume (Figure 6C), resulting in final yields of well-ordered trimers ranging from 2–4.5 mgs per liter of transfected 293F cells (Figure S5). Trimer stability, measured by DSC and reported as the T_m , ranged from 66°C to 77°C, displaying single narrow symmetric thermal transition curves that are characteristic of a homogeneous molecular species (Figure S6). Recognition of trimer-preferring V2-apex bNAbs (PGT145, PGDM1400, PG16, and VRC26), which bind one quaternary epitope per trimer, sug-

gested that these soluble trimers adopted native-like conformations. Binding affinities for these ligands were determined by BLI and ranged from 2 to 205 nM (Figure 6D and Table S1). Generally, higher affinities (< 100nM) for NFL TD CC+ trimers were associated with neutralization of the matched parental HIV strain by that same bNAb, while lower binding affinities (> 100nM) were associated with an inability of that

bNAb to neutralize the parental isolate from which the NFL was derived (Table S1). These results indicated that combining the HIV Env stabilizing elements (TD, CC, glycines, and the V3-FP mutations) facilitated the high-yield production of cross-clade stable soluble NFL trimers that were efficiently recognized by trimer-specific bNAbs.

DISCUSSION

Here, we described the design strategy and high-resolution crystal structure of a stabilized, soluble, subtype C HIV-1 Env trimer, 16055 NFL TD CC (T569G). The NFL protomer structure was virtually identical to that of subtype A BG505 SOSIP.664 and subtype G X1193.c1 SOSIP.664 soluble trimers and to subtype B native JRFL Env Δ CT, despite Env amino-acid-sequence variability in different subtypes and despite the fact that the NFL Env subunits, gp120 and gp41, are covalently linked by a flexible peptide linker. The structural differences were limited to the variable apex regions V1, V2, V4, and V5 in gp120, HR1_N

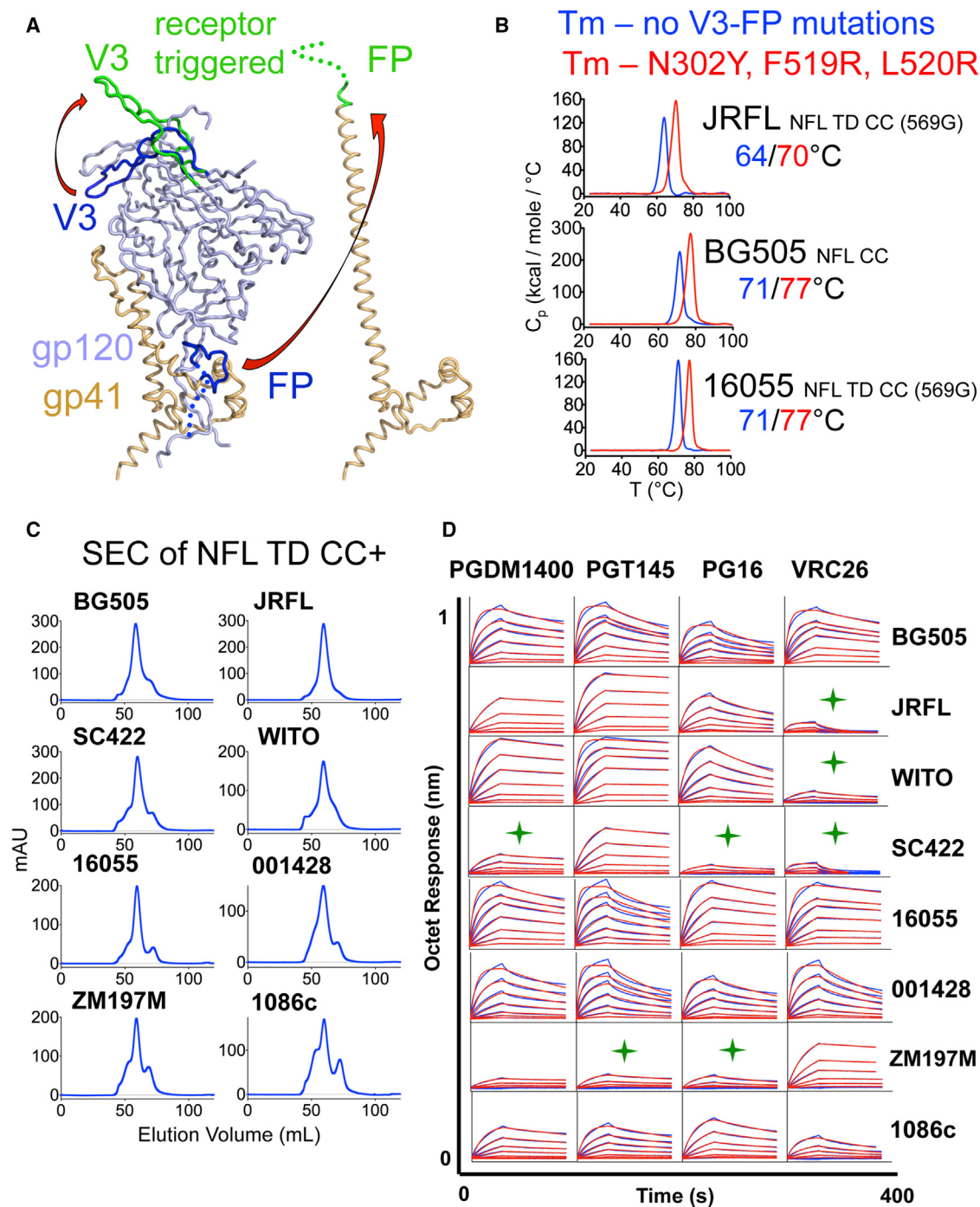

in gp41, and the glycan shield. Viral evolution, driven by immune selection pressure and accommodated by the error-prone reverse transcriptase, generates sequence diversity while conserving the key functional regions (receptor binding, fusion) that permit viral entry. Type I fusion proteins, such as the HIV-1 Env, mediate transfer of viral genetic material across the virus and host cell membranes to initiate replication. To carry out this vital function, fusion proteins adopt a highly metastable conformation in their pre-fusion state. Upon binding of receptor (and co-receptor), and sometimes exposure to low pH in endosomal compartments (e.g., influenza virus), large-scale Env conformational rearrangements are triggered, leading initially to extension of the long central helix (HR1_C) that propels the fusion peptide toward and into the target cell. This state subsequently collapses to the post-fusion six-helix bundle, mediating viral-to-cell-membrane fusion. We made helix-disrupting glycine substitutions in strategically located gp41 residues that would disfavor such transitions. We selected junctional sites that transition from coils to helices in the post-fusion conformation to disrupt their helical potential. In doing so, we presumably decreased Env-free energy required to transition to the fusion intermediate, thereby favoring instead the pre-fusion state. The targeted T569G mutation was instrumental in improving the homogeneity of the soluble 16055 NFL TD CC trimer, perhaps by decreasing the propensity of gp41 to “spring” to undesired post-fusion conformations. The modified 16055 NFL generated diffraction-quality crystals that enabled structure determination.

The protein engineering in this study was tailored to generate soluble mimics of the HIV-1 envelope glycoprotein—in this case, the NFL trimer—but likely will work in the SOSIP platform, as we showed for the TD CC design elements (Guenaga et al., 2015b). The combination of stabilizing elements (TD, CC, and V3-FP mutations) and glycine substitutions allowed us to generate stable and homogeneous soluble mimics of Env derived from diverse HIV strains that can be used for both candidate immunogens and for structural studies. More importantly, the glycine modifications and other elements involved in the stabilization could be applied to other viral fusion proteins that undergo large conformational changes in transitioning from a pre-fusion to a post-fusion state. Another recent successful strategy has been to truncate the N-terminal HR1 region by substitution of a shorter connecting region that increases its stability and expression (Kong et al., 2016). In contrast, our strategy here focused on retaining the length of the N-terminal HR1 region and applying targeted glycine substitutions to inhibit helix extension and the subsequent “spring-loaded” gp41 rearrangement of its individual helices in HR1 and HR2 to a more stable six-bundle helical structure for fusion. Targeted substitution of helix-disrupting glycines disfavors formation of intermediate, receptor-activated forms of the viral envelope protein, increasing native-like trimer yields and homogeneity. The well-ordered 16055 NFL clade C trimers described here were evaluated as immunogens following vaccination of NHPs, as reported in an accompanying manuscript. This study found that the 16055 NFL TD CC trimers, when arrayed at high-density on liposomes, induced autologous tier 2 neutralizing antibodies, targeting the Env V2 apical region (Martinez-Murillo et al., 2017).

STAR★METHODS

Detailed methods are provided in the online version of this paper and include the following:

- **KEY RESOURCES TABLE**
- **CONTACT FOR REAGENT AND RESOURCE SHARING**
- **METHOD DETAILS**
  - Design of NFL TD CC+ trimer constructs
  - Expression and purification of soluble proteins
  - Sample preparation for crystallization
  - Crystallization and data collection
  - Structure determination and refinement
  - Differential scanning calorimetry
  - Bio-Layer interferometry binding analysis
  - Electron microscopy and data collection
  - Electron microscopy data processing
  - Immunoprecipitation
- **DATA AND SOFTWARE AVAILABILITY**

SUPPLEMENTAL INFORMATION

Supplemental Information includes six figures and one table and can be found with this article online at <http://dx.doi.org/10.1016/j.immuni.2017.04.014>.

AUTHOR CONTRIBUTIONS

Project design by J.G., F.G., R.T.W., and I.A.W.; protein engineering and design by J.G. and R.T.W.; X-ray work and analysis by F.G., R.L.S., and I.A.W.; EM work by N.V. and A.B.W.; protein expression and purification J.G., V.D., B.C., and B.H.; DSC analysis by J.G. and B.H. Manuscript written or edited by J.G., F.G., A.B.W., R.T.W., and I.A.W.

ACKNOWLEDGMENTS

This work was supported by the International AIDS Vaccine Initiative Neutralizing Antibody Center and Collaboration for AIDS Vaccine Discovery (CAVD OPP1084519) (J.G., I.A.W., A.B.W., and R.T.W.). IAVI's work is made possible by generous support from many donors, including the following: the Bill & Melinda Gates Foundation; the Ministry of Foreign Affairs of Denmark; Irish Aid; the Ministry of Finance of Japan; the Ministry of Foreign Affairs of the Netherlands; the Norwegian Agency for Development Cooperation (NORAD); the United Kingdom Department for International Development (DFID), and the United States Agency for International Development (USAID). The full list of IAVI donors is available at www.iavi.org. We are very grateful to H. Tien for crystallization screening. X-ray data sets were collected at the GM/CA@APS-23ID-D beamline, which has been funded in whole or in part with Federal funds from the National Cancer Institute (ACB-12002) and the National Institute of General Medical Sciences (AGM-12006). This research used resources of the Advanced Photon Source (APS), a U.S. Department of Energy (DOE) Office of Science User Facility operated for the DOE Office of Science by Argonne National Laboratory under Contract No. DE-AC02-06CH11357. This work was supported by the Scripps Center for HIV/AIDS Vaccine Immunology and Immunogen Discovery (CHAVI-ID) UM1 AI00663 (A.B.W., I.A.W., and R.T.W.), by NIH P01 HIVRAD AI104722 (R.T.W.), by NIH R56 AI084817 (I.A.W.) and by the Joint Center of Structural Genomics (JCSG) funded by the NIH NIGMS Protein Structure Initiative U54 GM094586 (I.A.W.).

Received: December 13, 2016

Revised: March 16, 2017

Accepted: April 25, 2017

Published: May 16, 2017

REFERENCES

- Adams, P.D., Afonine, P.V., Bunkóczi, G., Chen, V.B., Davis, I.W., Echols, N., Headd, J.J., Hung, L.W., Kapral, G.J., Grosse-Kunstleve, R.W., et al. (2010). PHENIX: a comprehensive Python-based system for macromolecular structure solution. *Acta Crystallogr. D Biol. Crystallogr.* **66**, 213–221.
- Berger, E.A., Lifson, J.D., and Eiden, L.E. (1991). Stimulation of glycoprotein gp120 dissociation from the envelope glycoprotein complex of human immunodeficiency virus type 1 by soluble CD4 and CD4 peptide derivatives: implications for the role of the complementarity-determining region 3-like region in membrane fusion. *Proc. Natl. Acad. Sci. USA* **88**, 8082–8086.
- Binley, J.M., Sanders, R.W., Clas, B., Schuelke, N., Master, A., Guo, Y., Kajumo, F., Anselma, D.J., Maddon, P.J., Olson, W.C., and Moore, J.P. (2000). A recombinant human immunodeficiency virus type 1 envelope glycoprotein complex stabilized by an intermolecular disulfide bond between the gp120 and gp41 subunits is an antigenic mimic of the trimeric virion-associated structure. *J. Virol.* **74**, 627–643.
- Buzon, V., Natrajan, G., Schibli, D., Campelo, F., Kozlov, M.M., and Weissenhorn, W. (2010). Crystal structure of HIV-1 gp41 including both fusion peptide and membrane proximal external regions. *PLoS Pathog.* **6**, e1000880.
- Colman, P.M., and Lawrence, M.C. (2003). The structural biology of type I viral membrane fusion. *Nat. Rev. Mol. Cell Biol.* **4**, 309–319.
- Crooks, E.T., Tong, T., Chakrabarti, B., Narayan, K., Georgiev, I.S., Menis, S., Huang, X., Kulp, D., Osawa, K., Muranaka, J., et al. (2015). Vaccine-Elicited Tier 2 HIV-1 Neutralizing Antibodies Bind to Quaternary Epitopes Involving Glycan-Deficient Patches Proximal to the CD4 Binding Site. *PLoS Pathog.* **11**, e1004932.
- Eckert, D.M., and Kim, P.S. (2001). Mechanisms of viral membrane fusion and its inhibition. *Annu. Rev. Biochem.* **70**, 777–810.
- Elsiger, M.A., Deacon, A.M., Godzik, A., Lesley, S.A., Wooley, J., Wüthrich, K., and Wilson, I.A. (2010). The JCSG high-throughput structural biology pipeline. *Acta Crystallogr. Sect. F Struct. Biol. Cryst. Commun.* **66**, 1137–1142.
- Emsley, P., and Cowtan, K. (2004). Coot: model-building tools for molecular graphics. *Acta Crystallogr. D Biol. Crystallogr.* **60**, 2126–2132.
- Garces, F., Sok, D., Kong, L., McBride, R., Kim, H.J., Saye-Francisco, K.F., Julien, J.P., Hua, Y., Cupo, A., Moore, J.P., et al. (2014). Structural evolution of glycan recognition by a family of potent HIV antibodies. *Cell* **159**, 69–79.
- Garces, F., Lee, J.H., de Val, N., de la Peña, A.T., Kong, L., Puchades, C., Hua, Y., Stanfield, R.L., Burton, D.R., Moore, J.P., et al. (2015). Affinity maturation of a potent family of HIV antibodies is primarily focused on accommodating or avoiding glycans. *Immunity* **43**, 1053–1063.
- Gristick, H.B., von Boehmer, L., West, A.P., Jr., Schamber, M., Gazumyan, A., Golijanin, J., Seaman, M.S., Fätkenheuer, G., Klein, F., Nussenzweig, M.C., and Bjorkman, P.J. (2016). Natively glycosylated HIV-1 Env structure reveals new mode for antibody recognition of the CD4-binding site. *Nat. Struct. Mol. Biol.* **23**, 906–915.
- Guenaga, J., de Val, N., Tran, K., Feng, Y., Satchwell, K., Ward, A.B., and Wyatt, R.T. (2015a). Well-ordered trimeric HIV-1 subtype B and C soluble spike mimetics generated by negative selection display native-like properties. *PLoS Pathog.* **11**, e1004570.
- Guenaga, J., Dubrovskaya, V., de Val, N., Sharma, S.K., Carrette, B., Ward, A.B., and Wyatt, R.T. (2015b). Structure-guided redesign increases the propensity of HIV Env to generate highly stable soluble trimers. *J. Virol.* **90**, 2806–2817.
- Huang, J., Kang, B.H., Pancera, M., Lee, J.H., Tong, T., Feng, Y., Imamichi, H., Georgiev, I.S., Chuang, G.Y., Druz, A., et al. (2014). Broad and potent HIV-1 neutralization by a human antibody that binds the gp41-gp120 interface. *Nature* **515**, 138–142.
- Julien, J.P., Cupo, A., Sok, D., Stanfield, R.L., Lyumkis, D., Deller, M.C., Klasse, P.J., Burton, D.R., Sanders, R.W., Moore, J.P., et al. (2013). Crystal structure of a soluble cleaved HIV-1 envelope trimer. *Science* **342**, 1477–1483.
- Julien, J.P., Lee, J.H., Ozorowski, G., Hua, Y., Torrents de la Peña, A., de Taeye, S.W., Nieusma, T., Cupo, A., Yasmeen, A., Golabek, M., et al. (2015). Design and structure of two HIV-1 clade C SOSIP.664 trimers that increase the arsenal of native-like Env immunogens. *Proc. Natl. Acad. Sci. USA* **112**, 11947–11952.
- Klasse, P.J., Depetris, R.S., Pejchal, R., Julien, J.P., Khayat, R., Lee, J.H., Marozsan, A.J., Cupo, A., Cocco, N., Korzun, J., et al. (2013). Influences on trimerization and aggregation of soluble, cleaved HIV-1 SOSIP envelope glycoprotein. *J. Virol.* **87**, 9873–9885.
- Klasse, P.J., LaBranche, C.C., Ketkar, T.J., Ozorowski, G., Cupo, A., Pugach, P., Ringe, R.P., Golabek, M., van Gils, M.J., Guttman, M., et al. (2016). Sequential and simultaneous immunization of rabbits with HIV-1 envelope glycoprotein SOSIP.664 trimers from clades A, B and C. *PLoS Pathog.* **12**, e1005864.
- Kong, L., He, L., de Val, N., Vora, N., Morris, C.D., Azadnia, P., Sok, D., Zhou, B., Burton, D.R., Ward, A.B., et al. (2016). Uncleaved prefusion-optimized gp140 trimers derived from analysis of HIV-1 envelope metastability. *Nat. Commun.* **7**, 12040.
- Kwon, Y.D., Pancera, M., Acharya, P., Georgiev, I.S., Crooks, E.T., Gorman, J., Joyce, M.G., Guttman, M., Ma, X., Narpala, S., et al. (2015). Crystal structure, conformational fixation and entry-related interactions of mature ligand-free HIV-1 Env. *Nat. Struct. Mol. Biol.* **22**, 522–531.
- Lander, G.C., Stagg, S.M., Voss, N.R., Cheng, A., Fellmann, D., Pulokas, J., Yoshioka, C., Irving, C., Mulder, A., Lau, P.W., et al. (2009). Appion: an integrated, database-driven pipeline to facilitate EM image processing. *J. Struct. Biol.* **166**, 95–102.
- Lasky, L.A., Groopman, J.E., Fennie, C.W., Benz, P.M., Capon, D.J., Dowbenko, D.J., Nakamura, G.R., Nunes, W.M., Renz, M.E., and Berman, P.W. (1986). Neutralization of the AIDS retrovirus by antibodies to a recombinant envelope glycoprotein. *Science* **233**, 209–212.
- Lee, J.H., Ozorowski, G., and Ward, A.B. (2016). Cryo-EM structure of a native, fully glycosylated, cleaved HIV-1 envelope trimer. *Science* **351**, 1043–1048.
- Liu, J., Deng, Y., Dey, A.K., Moore, J.P., and Lu, M. (2009). Structure of the HIV-1 gp41 membrane-proximal ectodomain region in a putative prefusion conformation. *Biochemistry* **48**, 2915–2923.
- Luftig, M.A., Mattu, M., Di Giovine, P., Geleziunas, R., Hrin, R., Barbatto, G., Bianchi, E., Miller, M.D., Pessi, A., and Carfi, A. (2006). Structural basis for HIV-1 neutralization by a gp41 fusion intermediate-directed antibody. *Nat. Struct. Mol. Biol.* **13**, 740–747.
- Lyumkis, D., Julien, J.P., de Val, N., Cupo, A., Potter, C.S., Klasse, P.J., Burton, D.R., Sanders, R.W., Moore, J.P., Carragher, B., et al. (2013). Cryo-EM structure of a fully glycosylated soluble cleaved HIV-1 envelope trimer. *Science* **342**, 1484–1490.
- Martin, A.C. (1996). Accessing the Kabat antibody sequence database by computer. *Proteins* **25**, 130–133.
- Martinez-Murillo, P., Tran, K., Guenaga, J., Lindgren, G., Ådori, M., Feng, Y., Phad, G.E., Bernat, N.V., Bale, S., Ingale, J., et al. (2017). Particulate array of well-ordered HIV clade C Env trimers elicits neutralizing antibodies that display a unique V2 cap approach. *Immunity* **46**, this issue, 804–817.
- McCoy, L.E., van Gils, M.J., Ozorowski, G., Messmer, T., Briney, B., Voss, J.E., Kulp, D.W., Macauley, M.S., Sok, D., Pauthner, M., et al. (2016). Holes in the glycan shield of the native HIV envelope are a target of trimer-elicited neutralizing antibodies. *Cell Rep.* **16**, 2327–2338.
- Moore, J.P., McKeating, J.A., Weiss, R.A., and Sattentau, Q.J. (1990). Dissociation of gp120 from HIV-1 virions induced by soluble CD4. *Science* **250**, 1139–1142.
- Otwinowski, Z., and Minor, W. (1997). Processing of X-ray diffraction data collected in oscillation mode. *Methods Enzymol.* **276**, 307–326.
- Pancera, M., Majeed, S., Ban, Y.E., Chen, L., Huang, C.C., Kong, L., Kwon, Y.D., Stuckey, J., Zhou, T., Robinson, J.E., et al. (2010). Structure of HIV-1 gp120 with gp41-interactive region reveals layered envelope architecture and basis of conformational mobility. *Proc. Natl. Acad. Sci. USA* **107**, 1166–1171.
- Pancera, M., Zhou, T., Druz, A., Georgiev, I.S., Soto, C., Gorman, J., Huang, J., Acharya, P., Chuang, G.Y., Ofek, G., et al. (2014). Structure and immune recognition of trimeric pre-fusion HIV-1 Env. *Nature* **514**, 455–461.

- Pugach, P., Ozorowski, G., Cupo, A., Ringe, R., Yasmeen, A., de Val, N., Derking, R., Kim, H.J., Korzun, J., Golabek, M., et al. (2015). A native-like SOSIP.664 trimer based on an HIV-1 subtype B env gene. *J. Virol.* **89**, 3380–3395.
- Ratner, L., Fisher, A., Jagodzinski, L.L., Mitsuya, H., Liou, R.S., Gallo, R.C., and Wong-Staal, F. (1987). Complete nucleotide sequences of functional clones of the AIDS virus. *AIDS Res. Hum. Retroviruses* **3**, 57–69.
- Ringe, R.P., Yasmeen, A., Ozorowski, G., Go, E.P., Pritchard, L.K., Guttman, M., Ketas, T.A., Cottrell, C.A., Wilson, I.A., Sanders, R.W., et al. (2015). Influences on the design and purification of soluble, recombinant native-like HIV-1 envelope glycoprotein trimers. *J. Virol.* **89**, 12189–12210.
- Sanders, R.W., Vesanen, M., Schuelke, N., Master, A., Schiffner, L., Kalyanaraman, R., Paluch, M., Berkhout, B., Maddon, P.J., Olson, W.C., et al. (2002). Stabilization of the soluble, cleaved, trimeric form of the envelope glycoprotein complex of human immunodeficiency virus type 1. *J. Virol.* **76**, 8875–8889.
- Sanders, R.W., Derking, R., Cupo, A., Julien, J.P., Yasmeen, A., de Val, N., Kim, H.J., Blattner, C., de la Peña, A.T., Korzun, J., et al. (2013). A next-generation cleaved, soluble HIV-1 Env trimer, BG505 SOSIP.664 gp140, expresses multiple epitopes for broadly neutralizing but not non-neutralizing antibodies. *PLoS Pathog.* **9**, e1003618.
- Sharma, S.K., de Val, N., Bale, S., Guenaga, J., Tran, K., Feng, Y., Dubrovskaya, V., Ward, A.B., and Wyatt, R.T. (2015). Cleavage-independent HIV-1 Env trimers engineered as soluble native spike mimetics for vaccine design. *Cell Rep.* **11**, 539–550.
- Shu, W., Liu, J., Ji, H., Radigan, L., Jiang, S., and Lu, M. (2000). Helical interactions in the HIV-1 gp41 core reveal structural basis for the inhibitory activity of gp41 peptides. *Biochemistry* **39**, 1634–1642.
- Sorzano, C.O., Bilbao-Castro, J.R., Shkolnisky, Y., Alcorlo, M., Melero, R., Caffarena-Fernández, G., Li, M., Xu, G., Marabini, R., and Carazo, J.M. (2010). A clustering approach to multireference alignment of single-particle projections in electron microscopy. *J. Struct. Biol.* **171**, 197–206.
- Stewart-Jones, G.B., Soto, C., Lemmin, T., Chuang, G.Y., Druz, A., Kong, R., Thomas, P.V., Wagh, K., Zhou, T., Behrens, A.J., et al. (2016). Trimeric HIV-1-Env structures define glycan shields from clades A, B, and G. *Cell* **165**, 813–826.
- Suloway, C., Pulokas, J., Fellmann, D., Cheng, A., Guerra, F., Quispe, J., Stagg, S., Potter, C.S., and Carragher, B. (2005). Automated molecular microscopy: the new Leginon system. *J. Struct. Biol.* **151**, 41–60.
- Tan, K., Liu, J., Wang, J., Shen, S., and Lu, M. (1997). Atomic structure of a thermostable subdomain of HIV-1 gp41. *Proc. Natl. Acad. Sci. USA* **94**, 12303–12308.
- van Heel, M., Harauz, G., Orlova, E.V., Schmidt, R., and Schatz, M. (1996). A new generation of the IMAGIC image processing system. *J. Struct. Biol.* **116**, 17–24.
- Voss, N.R., Yoshioka, C.K., Radermacher, M., Potter, C.S., and Carragher, B. (2009). DoG Picker and TiltPicker: software tools to facilitate particle selection in single particle electron microscopy. *J. Struct. Biol.* **166**, 205–213.
- Wei, X., Decker, J.M., Wang, S., Hui, H., Kappes, J.C., Wu, X., Salazar-Gonzalez, J.F., Salazar, M.G., Kilby, J.M., Saag, M.S., et al. (2003). Antibody neutralization and escape by HIV-1. *Nature* **422**, 307–312.
- Weiss, M.S., and Hilgenfeld, R. (1997). On the use of the merging R factor as a quality indicator for X-ray data. *J. Appl. Cryst.* **30**, 203–205.

STAR★METHODS

KEY RESOURCES TABLE

REAGENT or RESOURCE	SOURCE	IDENTIFIER
Chemicals, Peptides, and Recombinant Proteins		
293fectin	Invitrogen	Cat#12347500
<i>Galanthus nivalis</i> agarose	Vector Labs	Cat#AL-1243
Endoglycosidase H	New England Biolabs	Cat#P0703L
R-Protein A Sepharose FF	GE Healthcare	Cat#17127903
1086c NFL TD	This paper	N/A
1086c NFL TD (T536G)	This paper	N/A
1086c NFL TD (L537G)	This paper	N/A
1086c NFL TD (L544G)	This paper	N/A
1086c NFL TD (L568G)	This paper	N/A
1086c NFL TD (T569G)	This paper	N/A
1086c NFL TD (N636G)	This paper	N/A
1086c NFL TD (Y638G)	This paper	N/A
JRFL NFL TD (T569G)	This paper	N/A
16055 NFL TD (T569G)	This paper	N/A
16055 NFL TD CC (T569G)	This paper	N/A
16055 NFL TD CC+	This paper	N/A
JRFL NFL TD CC+	This paper	N/A
BG505 NFL CC+	This paper	N/A
SC422 NFL TD CC+	This paper	N/A
WITO NFL TD CC+	This paper	N/A
1086c NFL TD CC+	This paper	N/A
001428 NFL TD CC+	This paper	N/A
ZM197 NFL TD CC+	This paper	N/A
Critical Commercial Assays		
QuikChange Lightning Multi Site-Directed Mutagenesis Kit	Agilent Technologies	Cat#210513
Deposited Data		
16055 NFL TD CC (T569G), 3.9 Å structure	This paper	PDB: 5UM8
Experimental Models: Cell Lines		
Human: FreeStyle 293F cells	Invitrogen	Cat#R79007
Human: 293S (GnTi ⁺) cells	ATCC	Cat#CRL-3022
Software and Algorithms		
ForteBio Data Analysis v7.1	ForteBio	http://www.fortebio.com/octet-software.html
Coot	Emsley and Cowtan, 2004	http://www2.mrc-lmb.cam.ac.uk/personal/pemsley/coot/
Leginon	Suloway et al., 2005	http://emg.nysbc.org/redmine/projects/leginon/wiki/Leginon_Homepage
Appion	Lander et al., 2009	http://emg.nysbc.org/redmine/projects/appion/wiki/Appion_Home
Clustering 2D Alignment	Sorzano et al., 2010	http://xmipp.cnb.csic.es/twiki/bin/view/Xmipp/WebHome
IMAGIC	van Heel et al., 1996	https://www.imagescience.de/imagic.html
PHENIX	Adams et al., 2010	https://www.phenix-online.org

(Continued on next page)

Continued

REAGENT or RESOURCE	SOURCE	IDENTIFIER
Other		
Anti-human IgG Fc Capture (AHC) Biosensors	ForteBio	Cat#18-5060
HiLoad 16/600 Superdex 200 pg column	GE Healthcare	Cat#28989335
Superdex 200 Increase 10/300 GL column	GE Healthcare	Cat#28990944
CaptureSelect LC-lambda (Hu) affinity matrix	ThermoFisher Scientific	Cat#084905

CONTACT FOR REAGENT AND RESOURCE SHARING

Further information and requests for resources and reagents should be directed to and will be fulfilled by the Lead Contact, Richard T. Wyatt (wyatt@scripps.edu).

METHOD DETAILS**Design of NFL TD CC+ trimer constructs**

HIV Env sequences (BG505, JRFL, SC422, WITO, 16055, 001428, ZM197M and 1086c (accession numbers DQ208458, U63632, AY835441, AY835451, EF117268, EF117266, DQ388515 and FJ444395, respectively) were modified to generate NFL TD CC+ soluble gp140 trimers as follows. The furin cleavage motif at the C terminus of gp120, “REKR,” was genetically deleted and replaced with two copies of the G₄S (GGGS) flexible linker, which covalently joins the C terminus of gp120 to the N terminus of gp41 (Sharma et al., 2015). A proline substitution at residue 559 was introduced by genetic means to facilitate trimer formation (Sanders et al., 2002). The gp140 sequences were genetically terminated at D664 (Klasse et al., 2013) followed by a G₄S linker, His₆ tag, and stop codon. The 16055 NFL protein construct used for the crystallization study had a stop codon after residue D664. All Env sequences except BG505 were modified with TD (BG505-trimer derived) residues and selected gp41 glycine substitutions to generate more stable and homogeneous NFL TD trimers (see Figure S5 for specific substitutions) (Guenaga et al., 2015b). Finally, all NFL TD+ constructs, including BG505, were genetically engineered with a C201-C433 disulfide (Guenaga et al., 2015b; Kwon et al., 2015) and V3-FP stabilizing mutations (Y302, R519 and R520). Substitutions in the Env-derived NFL glycoproteins were introduced via site-directed mutagenesis PCR using a QuikChange Lightning Multi Site-Directed Mutagenesis kit (Agilent Technologies) into expression plasmid DNAs. Substitutions were confirmed by sequencing (Genewiz).

Expression and purification of soluble proteins

Expression plasmids (CMV-R) encoding the NFL trimeric Env-derived glycoproteins were transfected in FreeStyle 293F or 293S cells using 293fectin (Invitrogen). Cell culture supernatants were harvested five days post transfection and the Env-derived proteins purified by lectin affinity chromatography (*Galanthus nivalis*, Vector Labs) followed by size exclusion chromatography (SEC) on a Superdex 200 16/60 or Superdex 200 10/300 GL (GE Healthcare). Similarly, all Fabs were produced by transient transfection in FreeStyle 293F and purified by affinity chromatography on a CaptureSelect LC-lambda column (ThermoFisher Scientific), followed by cation exchange chromatography and SEC on a Superdex 200 16/60.

Sample preparation for crystallization

Crystallization screening required the testing of many combinations of ligand-16055 NFL TD CC (T569G) trimer complexes in different conditions. Generally, trimers and antibody ligands were mixed in a 1:3.2 molar ratio before SEC purification of the complex. To decrease heterogeneity on the NFL trimer:ligand complexes, NFL trimer produced in 293S cells was complexed with antibodies and then deglycosylated using Endoglycosidase H (EndoH; New England Biolabs) in 200 mM NaCl, 50 mM sodium citrate pH 5.5 for 45 min at 37°C (Julien et al., 2013). Glycans interacting with or protected by interaction with the Fabs are generally preserved in their ‘native’ forms (in this case high mannose due to the cell line used for expression), but those accessible to EndoH are trimmed to the core GlcNAc moiety.

Crystallization and data collection

To determine the molecular architecture of the 16055 NFL TD CC (T569G) Env trimer, we analyzed the soluble NFL trimer in a ternary complex with Fabs PGT124 and 35O22. These Fabs maintain the native trimer conformation and facilitate crystal packing (Garces et al., 2015). The purified ternary complex was concentrated to ~12.2 mg/ml and screened in nearly 1000 crystallization conditions at 4 and 20°C using the IAVI/JCSG/TSRI CrystalMation robot (Rigaku) system at TSRI (Elslinger et al., 2010). Initial crystals were found in 0.2 M ammonium sulfate pH 4.5, 10% (v/v) glycerol, 20% (v/v) PEG 300 and 0.1 M phosphate-citrate pH 4.2. Optimized crystals were grown in 0.2 M ammonium sulfate, 10% (v/v) glycerol, 22% (v/v) PEG 300 and 0.1 M phosphate-citrate pH 4.03 and did not require

further cryoprotection (as the 10% glycerol in the crystallization condition was sufficient). The crystals were then stored by immediate flash cooling in liquid nitrogen. Data were collected at APS beamline 23-ID-D. Although we observed some diffraction to 3.5 Å, the final dataset was processed with HKL-2000 (Otwinowski and Minor, 1997) to 3.9 Å with an overall R_{sym} of 0.23% and 100% completeness in space group $P6_3$ with unit cell parameters: $a = b = 126.5$ Å, $c = 314.1$ Å (Table 1).

Structure determination and refinement

For the ternary complex, two previous structures were used for phasing by MR: a protomer of 35O22:BG505 SOSIP.664 (PDB: 5CEZ) and the high-resolution unliganded PGT124 Fab (PDB: 4R26). Model building was carried out using Coot (Emsley and Cowtan, 2004) and refinement with phenix.refine using reference model restraints calculated from the structure of 35O22:BG505 SOSIP.664 (PDB: 5CEZ) and the PGT124 Fab (PDB: 4R26) (Adams et al., 2010). The final R_{cryst} and R_{free} values are 27.4% and 31.8% (Table 1). For the Fabs, residues were numbered according to Kabat (Martin, 1996), and gp120/gp41 numbered following the HXBc2 system (Ratner et al., 1987).

Differential scanning calorimetry

The thermal transition points (T_m) of the NFL trimeric proteins were determined by differential scanning calorimetry (DSC) using a MicroCal VP-Capillary DSC instrument (Malvern). Prior to the DSC melting scan, the protein samples were extensively dialyzed in PBS, pH 7.4, and the concentrations were adjusted to 0.25 mg/ml. The dialysis buffer was used as the reference solution. The DSC experiments were done at a scanning rate of 1 K/min under 3.0 atmospheres of pressure. DSC data were analyzed after buffer correction, normalization, and baseline subtraction using MicroCal VP-Capillary DSC analysis software provided by the manufacturer.

Bio-Layer interferometry binding analysis

All binding measurements were carried out on an Octet Red instrument (ForteBio) with IgGs immobilized on anti-human IgG Fc capture sensors (ForteBio). The NFL trimers were assessed as free analytes in solution (PBS pH 7.4). For measurement of the V2-Apex bNAbs kinetic parameters, the analytes (NFL trimers) were concentrated to 800nM and then serially diluted 1:2 to a final concentration of 12.5nM. Association and dissociation times were 2 and 4 min respectively. Data were analyzed using the ForteBio analysis software version 7.1 (ForteBio) and the kinetic parameters were calculated using a global fit 1:1 model.

Electron microscopy and data collection

Following lectin purification, the NFL TD proteins were analyzed by negative-stain EM. A 3 μ L aliquot containing ~ 0.03 mg/mL of the sample was applied for 15 s onto a carbon-coated 400 Cu mesh grid that had been glow discharged at 15 mA for 30 s, then negatively stained with 2% uranyl formate for 50 s. Data were collected using a FEI Tecnai Spirit electron microscope operating at 120 kV, with an electron dose of ~ 30 e $^{-}$ /Å ^{2} and a magnification of 52,000 x that resulted in a pixel size of 2.05 Å at the specimen plane. Images were acquired with a Tietz 4k $\times$ 4k TemCam-F416 CMOS camera using a nominal defocus of 1000 nm and the Leginon package (Suloway et al., 2005).

Electron microscopy data processing

Particles were picked automatically using DoG Picker and put into a particle stack using the Appion software package (Lander et al., 2009; Voss et al., 2009). Reference-free, two-dimensional (2D) class averages were calculated using particles binned by two via the iterative msa/mra Clustering 2D Alignment and IMAGIC software systems and sorted into classes (Sorzano et al., 2010; van Heel et al., 1996). To analyze the quality of the trimers (closed, partially open, or non-native like trimers), reference-free 2D class averages were examined by visual inspection using the same metrics as previously described (Pugach et al., 2015).

Immunoprecipitation

Supernatants containing overexpressed NFL proteins were subjected to immunoprecipitation analysis to determine the relative amount of native-like trimers within the mixture. Briefly, 20 μ L of rProtein A Sepharose beads (GE Healthcare) were added to an Eppendorf tube, washed twice with PBS, and resuspended in 500 μ L of PBS. Selected antibodies were added to the tubes containing the agarose beads (5 μ g per antibody in separate tubes). The mixture was rocked for 30 min at 4°C and then washed twice with 500mM NaCl PBS to removed unbound antibody. Supernatants were aspirated without disturbing the beads and 1 mL of NFL protein containing filtered supernatant was added to each tube. After one-hour incubation at RT, the tubes were centrifuged at 1000 g for 5 min and the supernatant containing unbound protein was discarded. The protein A-agarose pellets containing the bound antibody-Env complexes were washed twice with 1 mL of PBS and then resuspended in 20 μ L of SDS-PAGE loading buffer. The protein complexes were resolved over an SDS-PAGE 4%–12% Bis Tris NuPAGE gel (Invitrogen) for 50 min at 200 V.

DATA AND SOFTWARE AVAILABILITY

The 16055 NFL TD CC (T569G) crystal structure has been deposited to the RCSB Protein Data Bank with accession number PDB: 5UM8.

Supplemental Information

**Glycine Substitution at Helix-to-Coil Transitions
Facilitates the Structural Determination of
a Stabilized Subtype C HIV Envelope Glycoprotein**

Javier Guenaga, Fernando Garces, Natalia de Val, Robyn L. Stanfield, Viktoriya Dubrovskaya, Brett Higgins, Barbara Carrette, Andrew B. Ward, Ian A. Wilson, and Richard T. Wyatt

A

C

Figure 1 consists of two vertically stacked plots showing the heat capacity C_p as a function of temperature T for poly(2-vinylpyridine). Both plots share a common x-axis representing temperature T in degrees Celsius ($^{\circ}\text{C}$), ranging from 20 to 100 with major ticks every 20 units. The top plot's y-axis is C_p in $\text{J} / \text{mole} / ^{\circ}\text{C}$, ranging from 0 to 200 with major ticks every 100 units. It features an orange curve with a sharp endothermic peak labeled 67.3°C in orange text. The bottom plot's y-axis is C_p in $\text{kcal} / \text{mole} / ^{\circ}\text{C}$, also ranging from 0 to 200 with major ticks every 100 units. It features a purple curve with a sharp endothermic peak labeled 67.7°C in purple text. Both curves show a baseline that is nearly constant at lower temperatures and rises sharply at the transition temperature before returning to a new baseline at higher temperatures.

B

Single G

VRC03 VRC01

TD

544

568

569

636

Double G

VRC03 VRC01

568

544

568

636

569

544

569

636

544

636

Triple G

VRC03 VRC01

568

544

636

569

544

636

SEC Chromatogram

66.3 kDa

0 60 80 100

Figure S1. Effect of glycine substitutions on NFL TD proteins. Related to Figure 1. (A) EM negative-stain 2D class averages of 1086c NFL TD trimer variants carrying selected gp41 glycine substitutions. The EM-derived frequencies of native-like forms of the trimer variants are shown as percentages of the total purified protein. (B) SDS-gel of 1086c NFL TD proteins immunoprecipitated with the trimer-independent CD4bs bNAb VRC01 to control for expression and with the trimer-preferring bNAb VRC03 to indicate the presence of native-like, well-ordered trimers from cell supernatants containing the transiently expressed trimer variants possessing the indicated glycine substitutions. (C) SEC and DSC profiles corresponding to the 16055 NFL TD proteins with glycine substitutions at positions 568 and 569.

FIG S2

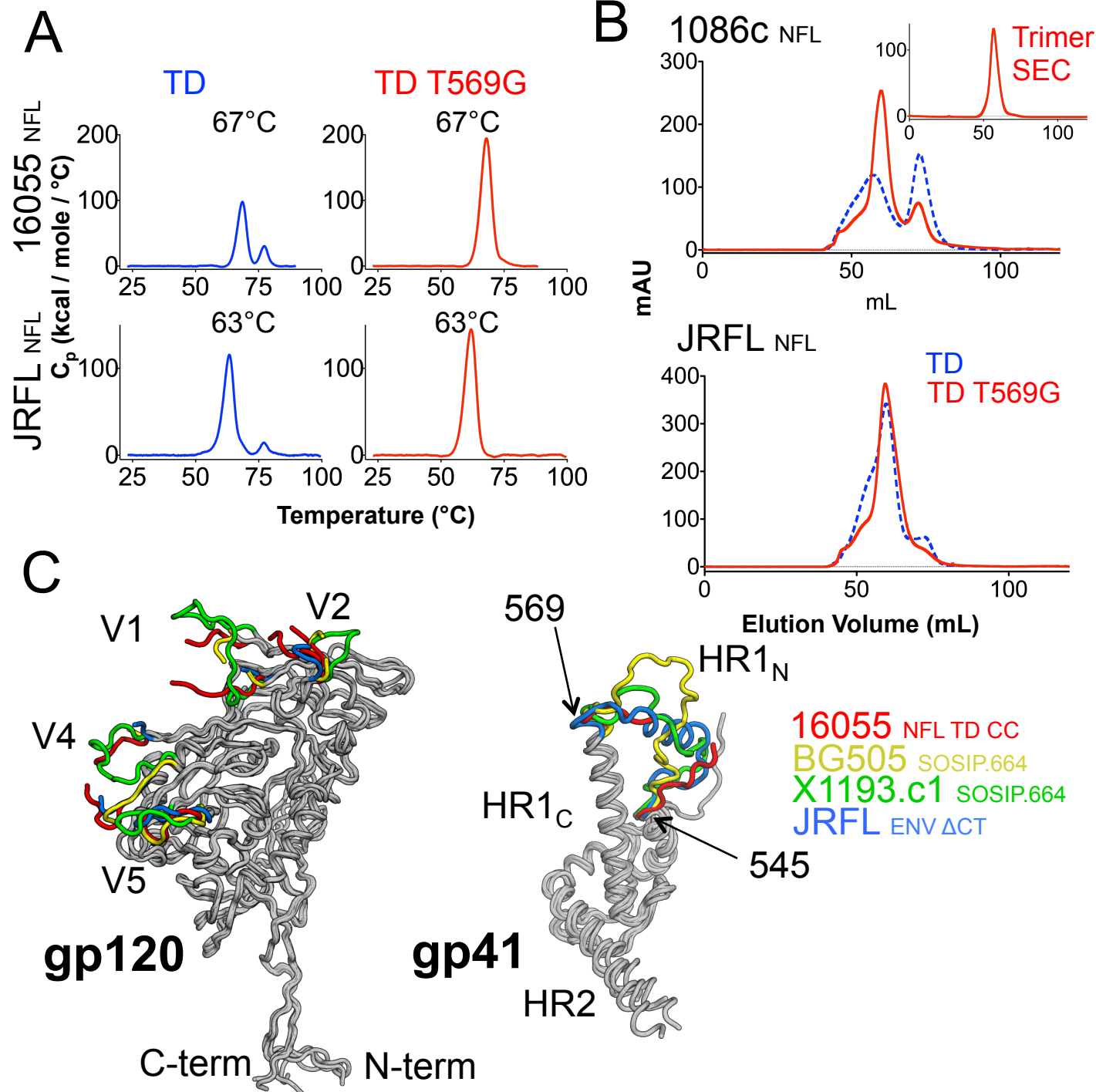

Figure S2. Stability and homogeneity of NFL TD proteins with the T569G mutation and Env regions with structural variation. Related to Figures 1 and 2. **(A)** DSC thermal transition curves of the 16055 and JRFL NFL TD trimers with and without the T569G mutation. **(B)** SEC profiles of HIV-1 subtype C- and B-derived 1086c NFL TD and JRFL NFL TD trimers with and without the T569G mutation. Shown in the upper right is the SEC profile corresponding to a re-run of the trimer fractions. **(C)** Superimposition of structures of Env subunits corresponding to four different HIV strains with differences highlighted in color [(16055, red), (BG505, yellow PDB 5CEZ), (X1193.c1, green PDB 5FYJ), and (JRFL, blue PDB 5FUU)].

FIG S3

A

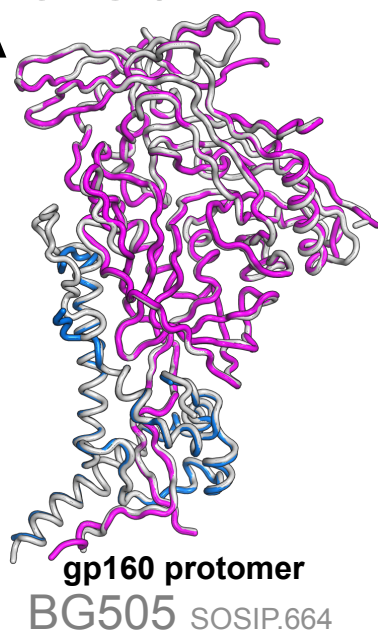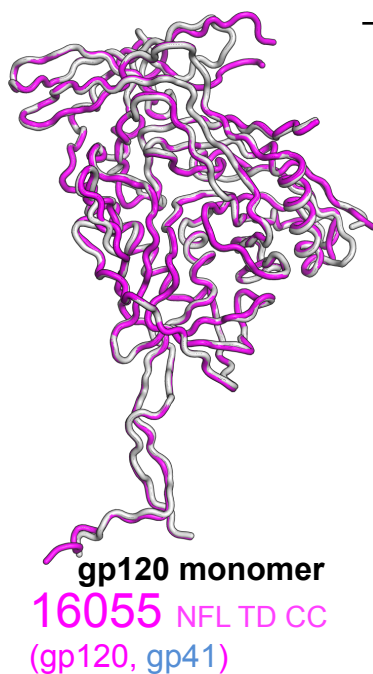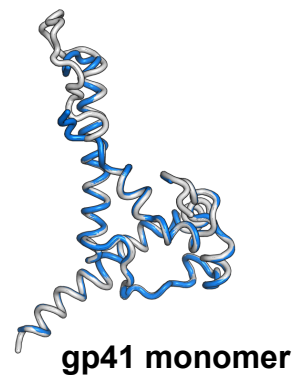

Superimpositions	R.M.S.D.
gp160 protomer	0.7 Å
gp120 monomer	0.6 Å
gp41 monomer	0.6 Å

B

TD residues

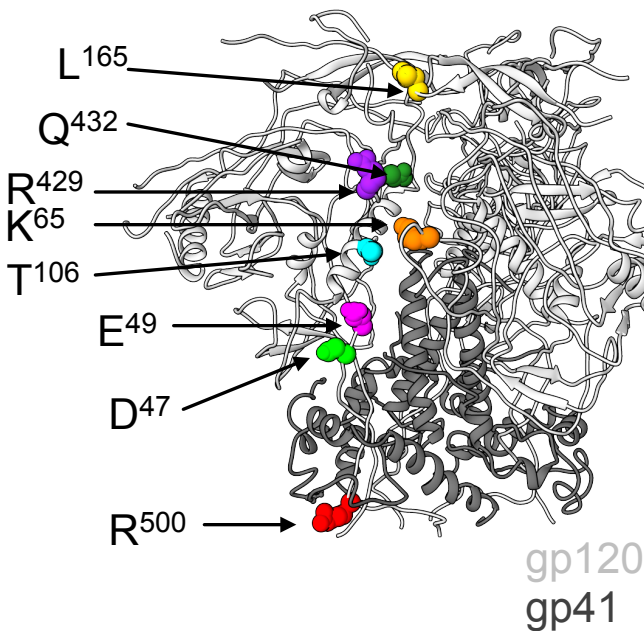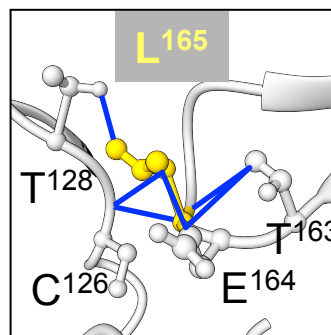

Apex domain

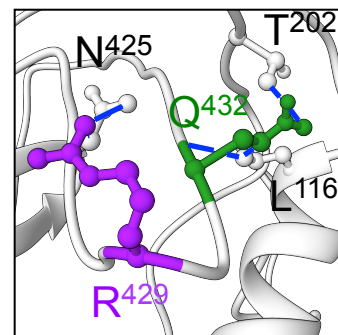

Pre-bridging sheet

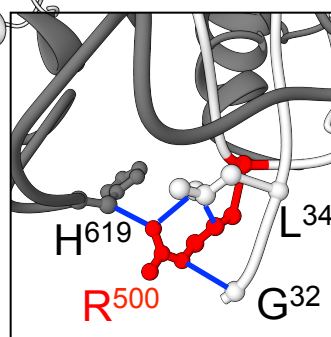

gp120-gp41
interface

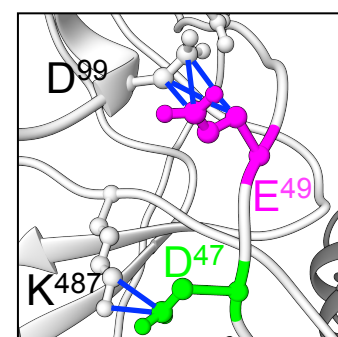

gp120 N-
terminus

Figure S3. Structural analysis of 16055 NFL TD CC (T569G). Related to Figure 2. (A) Superimposition of the gp160 protomers (left), gp120 subunits (middle), and gp41 subunits (right) from the PGT124/35O22-bound 16055 and the PGT121/35O22-bound BG505 crystal structures with Ca RMSD measurements. (B) Cartoon representation of the 16055 NFL TD CC trimer with TD residues represented as colored spheres (left). Close-up view of specific TD residues and their contacts (right).

FIG S4

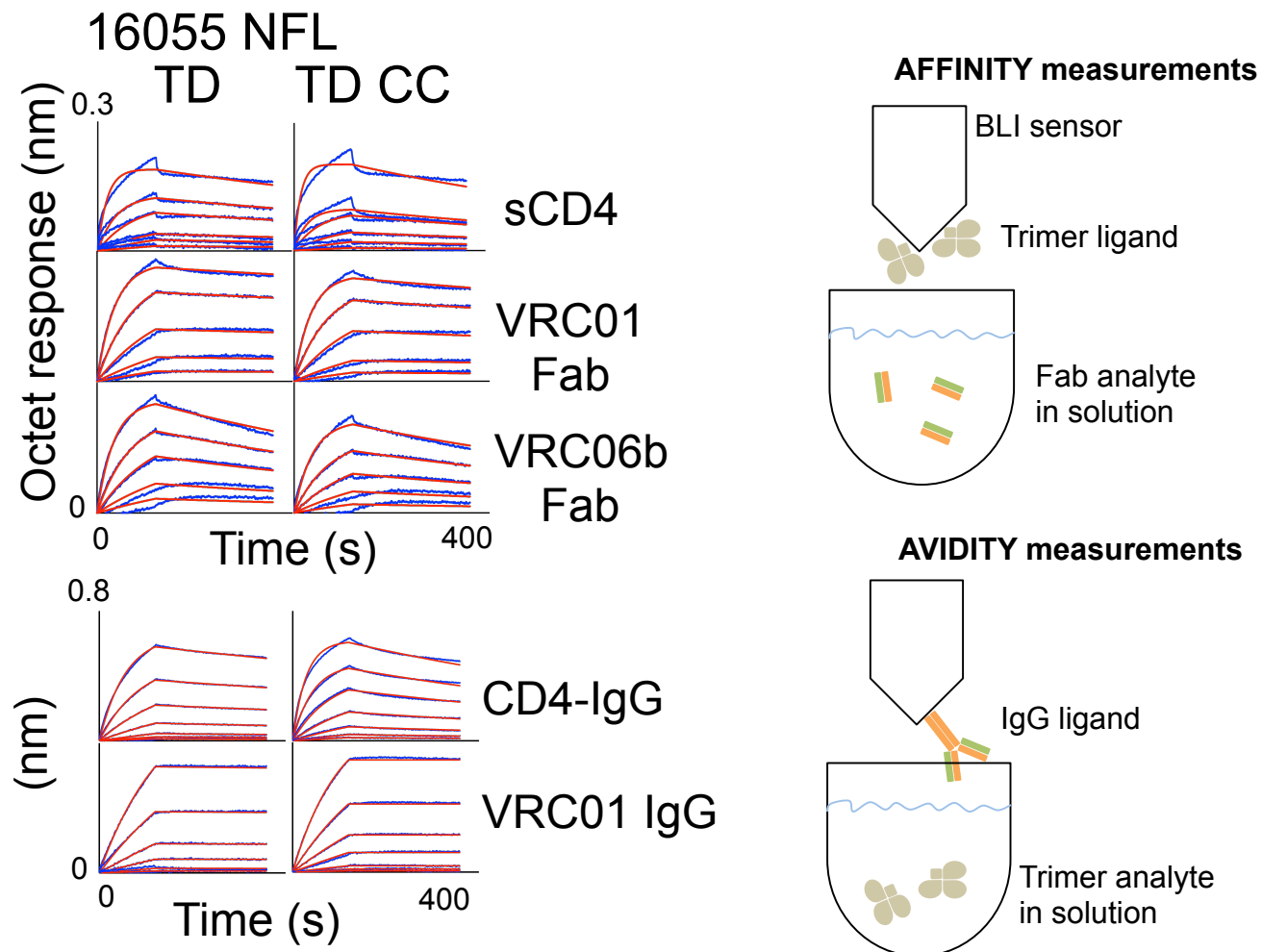

Analyte	Ligand	K _D (nM)	k _{on} (1/Ms)	k _{off} (1/s)
sCD4	TD	54	1.6 × 10 ⁴	8.7 × 10 ⁻⁴
	TD CC	54	2.2 × 10 ⁴	1.2 × 10 ⁻³
VRC01 Fab	TD	30	8.5 × 10 ³	2.5 × 10 ⁻⁴
	TD CC	44	9.0 × 10 ³	4.0 × 10 ⁻⁴
VRC06b Fab	TD	270	4.4 × 10 ³	1.2 × 10 ⁻³
	TD CC	282	4.0 × 10 ³	1.1 × 10 ⁻³
Trimer TD	CD4-IgG	25	2.2 × 10 ⁴	5.5 × 10 ⁻⁴
Trimer TD CC		25	4.3 × 10 ⁴	1.1 × 10 ⁻³
Trimer TD	VRC01 IgG	<1	8.9 × 10 ³	<1 × 10 ⁻⁵
Trimer TD CC		<1	1.1 × 10 ⁴	<1 × 10 ⁻⁵

AFFINITY

AVIDITY

Figure S4. BLI measurements of selected CD4bs ligands and NFL trimers. Related to Figure 5. BLI binding curves (blue) and Langmuir (1:1) model fits (red) for the interaction of monomeric analytes (four-domain-sCD4, VRC01 and VRC03 Fabs) with trimeric ligands, 16055 NFL TD (T569G) with and without 201-433CC. Avidity measurements were done with trimeric Env proteins as analytes in solution and bivalent ligands (IgG-CD4 and VRC01 IgG) immobilized on a biosensor. The K_D, k_{on} and k_{off} are tabulated below the Octet sensorgrams.

FIG S5

Yield and Design Elements of NFL TD CC+ Trimers

HIV Env<br>Seq ID	TD (BG505 Trimer Derived)														
	47	49	65	106	164	165	172	308	429	432	500	543	553	588	662
BG505	D	E	K	T	E	L	V	R	R	Q	R	N	S	R	A
JRFL	E-D	T-E	V-K	E-T	S-E	I-L	E-V	H-R	E-R	K-Q	K-R	L-N	N-S	G-R	E-A
SC422	E-D	K-E	K	E-T	A-E	I-L	T-V	T-R	E-R	K-Q	K-R	L-N	N-S	R	E-A
WITO	E-D	N-E	V-K	E-T	V-E	I-L	E-V	N-R	E-R	K-Q	R	L-N	S	K-R	E-A
16055	E-D	K-E	V-K	E-T	E	I-L	V	R	E-R	R-Q	A-R	Q	S	K	A
001428	E-D	R-E	V-K	E-T	E	I-L	A-V	R	E-R	R-Q	R	Q	S	K	A
ZM197M	E-D	K-E	V-K	E-T	E	V-L	Q-V	R	E-R	R-Q	S-R	Q	S	K	A
1086c	E-D	K-E	V-K	E-T	E	L	V	R	E-R	R-Q	E-R	Q	S	K	A

HIV Env<br>Seq ID	CC		G			V3-FP			Added Glycans	~YIELD (mg)<br>per Liter	Clade
	201	433	568	569	636	302	519	520			
BG505	I-C	A-C	L	T	S	N-Y	F-R	L-R	N332	2	A
JRFL	I-C	A-C	L	T-G	D	N-Y	F-R	L-R		4.5	
SC422	I-C	A-C	L	T-G	N	N-Y	F-R	L-R		4	
WITO	I-C	A-C	L	T-G	D-G	N-Y	F-R	L-R		3	B
16055	I-C	A-C	L	T-G	S-G	N-Y	F-R	L-R	N332	2.5	
001428	I-C	A-C	L-G	T	S-G	N-Y	F-R	L-R		2	
ZM197M	I-C	A-C	L-G	T	D-G	N-Y	I-R	L-R	N160 N295 N332	2	C
1086c	I-C	A-C	L-G	T	N-G	N-Y	F-R	L-R	N160	2	

Figure S5. Design elements of the NFL TD CC+ soluble trimers and yields. Related to Figure 6. Key elements of the NFL TD CC+ trimer design: the TD (or BG505 “Trimer Derived”) residues, the gp41 glycine substitutions that promote trimer formation, the engineered disulfide CC (I201C-A433C), and the V3-FP (N302Y, F519R and L520R) substitutions that contribute to an increase in soluble trimeric NFL Env stability.

FIG S6

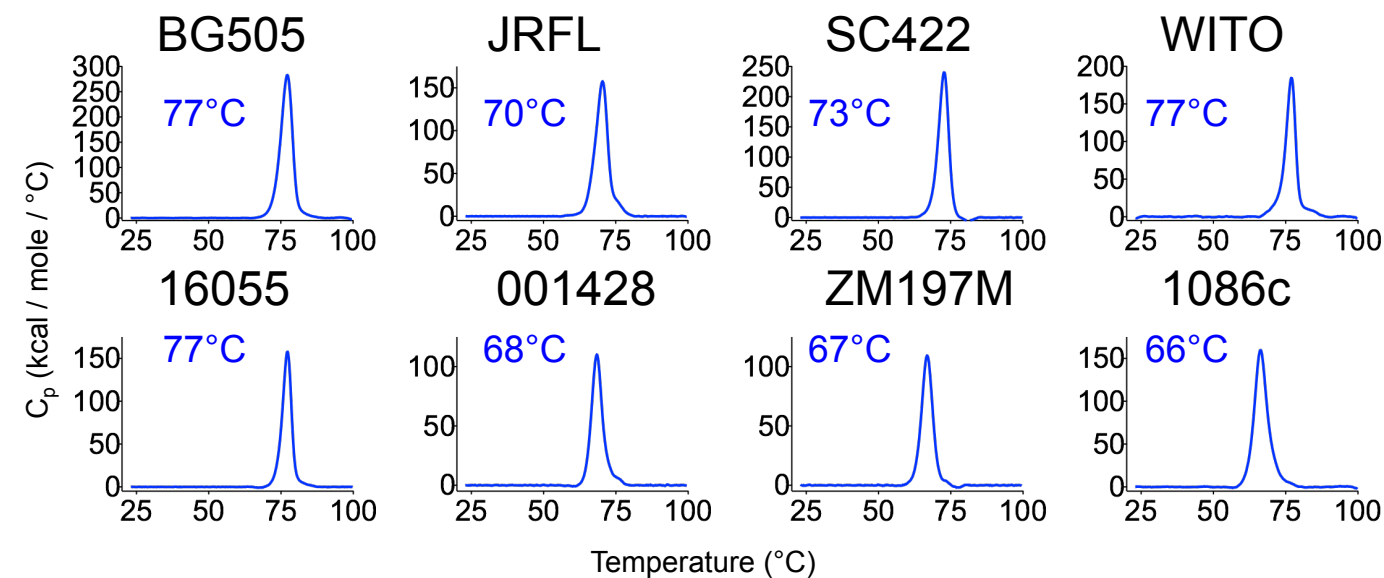

Figure S6. Stability of trimeric NFL proteins that include TD, CC, Glycines and V3-FP design elements. Related to Figure 6. DSC measurement of thermal transition midpoints of the NFL TD CC+ trimers following lectin and SEC purification.

Table S1.

**NFL<br>TD CC+**	**mAb**	**K_D<br>(nM)**	**k_{on}<br>(1/Ms) × 10⁴**	**k_{off}<br>(1/s) × 10⁻³**	**HIV-1<br>Neutralization<br>ug/mL**
BG505	PGDM1400	**20**	6.5	1.3	0.001
	PGT145	**22**	9.4	2.1	0.025
	PG16	**45**	7.1	3.2	0.005
	VRC26	**14**	8.2	1.1	0.001
JRFL	PGDM1400	**15**	3.2	0.5	*
	PGT145	**3**	7.2	0.2	0.064
	PG16	**40**	7.3	2.9	0.023
	VRC26	**107**	12.1	12.9	>50
WITO	PGDM1400	**13**	3.5	0.5	0.001
	PGT145	**2**	8.0	0.2	0.0004
	PG16	**62**	3.8	2.3	0.0005
	VRC26	**108**	2.7	2.9	>50
SC422	PGDM1400	**53**	2.0	1.1	0.550
	PGT145	**24**	2.3	0.5	0.0004
	PG16	**156**	1.8	2.9	3.18
	VRC26	**205**	7.4	15	>50
16055	PGDM1400	**15**	7.6	1.2	0.001
	PGT145	**18**	13	2.3	0.008
	PG16	**17**	4.5	0.8	0.002
	VRC26	**8**	5.9	0.5	0.004
1086c	PGDM1400	**40**	2.6	1.0	*
	PGT145	**41**	4.1	1.7	*
	PG16	**55**	2.9	1.6	*
	VRC26	**78**	3.7	2.9	*
001428	PGDM1400	**32**	6.1	1.9	0.001
	PGT145	**26**	12	3.2	0.0006
	PG16	**44**	5.1	2.2	0.023
	VRC26	**43**	7.3	3.1	0.010
ZM197M	PGDM1400	**29**	2.1	0.6	0.029
	PGT145	**67**	2.2	1.5	0.523
	PG16	**124**	2.2	2.7	6.67
	VRC26	**18**	2.0	0.3	0.002

* Not determined

Table S1. Kinetic parameters of the NFL trimers with trimer-preferred V2-apex bNAbs and HIV-1 neutralization. Related to figure 6. The above table displays the affinity parameters measured by bio-layer light interferometry of the NFL trimers with trimer-preferred V2-apex bNAbs. Last column (right) shows the bNAb capacities to neutralize the HIV-1 strains from which the NFL trimers were derived as IC50 (ug/mL) values.
